# Genome Mining and Structural Study of Cathelicidins Across Chiroptera Species

**DOI:** 10.1155/bri/5461549

**Published:** 2025-09-23

**Authors:** Manuel R. López, Beatriz González-Almécija, Francisco Farrais-Solana, Andrea Otazo-Pérez, Sergio González-Acosta, Patricia Asensio-Calavia, Antonio Morales-delaNuez, José-Manuel Pérez de la Lastra

**Affiliations:** ^1^Macromolecules Biotechnology Research Group, Institute of Natural Products and Agrobiology-Spanish National Research Council (IPNA-CSIC), San Cristóbal de La Laguna, Spain; ^2^Doctoral and Postgraduate Studies School, University of La Laguna, San Cristóbal de La Laguna, Spain; ^3^Animal Production and Biotechnology Group, Institute of Animal Health and Food Safety, University of Las Palmas de Gran Canaria, Las Palmas de Gran Canaria, Spain

**Keywords:** antimicrobial peptides, bats, cathelicidin, chiroptera, mining

## Abstract

The authors explored the genomic landscape of bat cathelicidins, critical proteins in the vertebrate innate immune system, across 41 bat species, which are notable for their high microbial tolerance. An array of bioinformatics tools, complemented by manual mining, were used to identify the curated cathelicidin sequences. Particular attention was given to the fourth exon, which is responsible for generating the biologically active peptide. This comprehensive exploration led to the identification of 72 annotated complete cathelicidins, with 44 being newly discovered. These 72 complete cathelicidin sequences, along with 7 incomplete and 4 nonfunctional sequences, result in a total of 78 peptides, 53 of which were previously unknown. These bat cathelicidins are characterized by a cathelicidin domain pfam00666, and the consensus sequence derived from aligning these domains shows a high degree of conservation in the position of each amino acid across all bat species, suggesting that they possess only one type of cathelicidin. Notably, the three-dimensional structure of the bat cathelicidin family domain closely resembles the cystatin domain previously described in *Sus scrofa* protegrin 3. The phylogenetic tree constructed using the bat conserved domain cluster species according to their taxonomic order, indicating the potential utility of this sequence as taxonomic markers. Finally, the authors propose a peptide classification based on three-dimensional structures and the presence or absence of the CAP18 domain, proline-rich or arginine-rich sequences. This approach holds great promise for future research focussed on precisely identifying antimicrobial peptides, conducting structure–activity and mechanism-of-action relationship studies and enhancing our understanding of the immune defence mechanisms in bats.

## 1. Introduction

The World Health Organization (WHO) recognizes antimicrobial resistance as a major public health concern. According to a 2022 report by the organization, the European Union/European Economic Area (EU/EEA) experiences over 670,000 antibiotic-resistant bacterial infections annually, resulting in approximately 33,000 deaths. Consequently, the development of novel antimicrobial agents is essential to combat this growing threat in the future [1, 2].

In the last decades, a variety of peptides with antimicrobial activity, called antimicrobial peptides (AMPs) or host defence peptides (HDPs), have been described in many plants [3], insects [4] and vertebrate species [5]. Among these peptides, those derived from cathelicidins, which are found in vertebrates, are extensively investigated [6–8]. They are recognized for their crucial role not only as antimicrobial agents but also as immunomodulators within the innate immune system [9–11]. The genes responsible for encoding cathelicidins consist of four exons [12]. Positioned towards the amino-terminal end, the protein features a notably conserved sequence enclosed by a signal peptide and a domain known as the cathelin conserved domain, which is encoded by exons I, II and III. Exon IV encompasses the region encoding the processing site and the sequence for the peptide. This sequence represents the sole hypervariable region in the gene [12–14].

However, cathelicidin itself exists as the inactive precursor, referred to as prepropeptide [15, 16]. To generate an active HDP, a signal peptidase plays a crucial role removing the signal sequence, giving rise to the propeptide. After the secretion of the propeptide, a series of specific enzymes are involved in additional processing to release the active HDP. In certain mammals, it has been described that the enzyme responsible for this cleavage is elastase, which is found in neutrophils [17]. Depending on the type of animal, the resulting peptide varies in size and sequence [14] and possess the capability to interact with the microorganism membranes, proteins and even DNA, thereby demonstrating activity against them [5, 18, 19].

Bats (Chiroptera order) constitute a diverse group that accounts for approximately 20.6% of the Mammalia taxa with 1116 species [20]. This taxonomic order exhibits distinctive biological features, such as laryngeal echolocation, the capacity for flight and remarkable longevity. Moreover, these animals act as reservoirs for a multitude of zoonotic viruses, parasites, fungi and bacteria, a noteworthy aspect considering their apparent absence of pathological signs associated with these diseases and infections [21, 22].

Previous investigations into bat cathelicidins have been conducted showing antimicrobial activity [23, 24]. However, challenges were encountered in precisely identifying their sequences and genomic locations, leading to the identification of inaccurate sequences [22]. This study aims to mine the genome to describe cathelicidin genes in bats by analysing their genomic positions, gene and protein structures and derived active peptides across all bat species archived in the NCBI database up to June 2023.

## 2. Materials and Methods

### 2.1. Probe Construction

The NCBI database was searched for bat cathelicidin sequences using the keywords ‘Cathelicidin' or ‘Cathelicidin-Like' and ‘Chiroptera'. The FASTA files containing the identified sequences were downloaded. A total of four sequences were randomly selected (Table 1(a)). The alignment of the four cathelicidins was performed using the CLUSTALW [25] and Jalview 2.11.3.0 software, resulting in a consensus sequence or probe (Table 1(b)).

### 2.2. Genome Mining

Using this probe, the authors conducted a genome mining analysis to search for cathelicidins across the species within the Chiroptera order, using the available assemblies (scaffolds, contigs or chromosomes) from each species in the NCBI genome database (until June 2023). By employing tblastn [26] with the probe, the authors effectively identified a group of sequences that exhibited significant alignment. Subsequently, the authors downloaded the genomic DNA (gDNA) sequences in FASTA format from the assemblies and selected the region (∼10^6^ base pair (bp)) containing the aligned sequence using the bioinformatics software Unipro UGENE 49.0 [27].

To precisely map the cathelicidins and identify the flanking genes within gDNA fragment, the authors utilized FGENESH, a gene prediction software developed by Softberry [28]. In this computational analysis, the authors introduced the gDNA sequence (previously selected with Unipro UGENE 49.0) into FGENESH, allowing us to accurately determine the gene positions and exon sizes along the provided sequence.

Finally, the authors used the NCBI's Conserved Domain Search Tool [29] to identify conserved domains in each cathelicidin amino acid sequence that the authors found during the genome mining process.

### 2.3. Alignment of Bat Cathelicidin Domain

The amino acid sequences of the conserved cathelicidin domain in FASTA format were aligned using the bioinformatic tool ClustalW [25] and summarized using Unipro UGENE v. 49.0 [27]. Using this software, the alignment of 72 bat cathelicidin domains was depicted in a per cent identity diagram, where the intensity of the blue colour indicates sequence similarity: the greater the similarity, the more intense the blue. This alignment resulted in a consensus sequence of 102 amino acids. Additionally, a histogram was employed, with each amino acid accompanied by a bar indicating the percentage of aligned sequences with that amino acid at that position, thus representing the dominant amino acid at each site. The consensus sequence was presented in uppercase only for positions conserved across all aligned domains.

### 2.4. Phylogenetic Analysis of Bat Conserved Region (Cathelin Domain)

The amino acid sequences of the corresponding cathelin domain from the putative bat cathelicidins were aligned using MUSCLE, and a phylogenetic tree was constructed using MEGA version 11.0.13 using maximum likelihood employing the Jones–Taylor–Thorton (JTT) amino acid substitution model (JTT + G + I). Nodal support in the unrooted trees was assessed through bootstrapping with 1000 replicates [30].

### 2.5. Repairing the Anomalous Sequences of the Fourth Exon

Bioinformatics tools used for gene prediction, occasionally mislocate the fourth exon or hypervariable domain in gDNA, leading to inaccurate peptide predictions of cathelicidins. To address this, the authors used a word processor to locate all exons, introns and the 5′ and 3′ untranslated regions (UTRs) within the gDNA sequences. When the fourth exon was mislocated or incorrect, the authors examined the sequence downstream from the third exon to the end of the gDNA in search of a stop codon or PolyA tail. Subsequently, the authors carefully examined all potential initiation and termination sequences for the presence of a possible third intron to precisely identify the initial nucleotides of the fourth exon. The candidate sequences were translated into the three reading frames, using the ExPASy translate tool [31]. The correct frame and exon were verified through comparative analysis with other cathelicidins using the alignment tools ClustalW [25]. This technique was systematically repeated with all available gDNA assemblies for the order Chiroptera in the NCBI database, available until June 2023.

### 2.6. Peptides Processing and Designation

It is known that neutrophil elastase processes cathelicidins in mammals, with valine being the most abundant elastase-sensitive residue [32]. Therefore, the authors identified the AMP as the sequence located after the first valine residue in the amino acid sequence of the fourth exon.

Peptide nomenclature followed a specific pattern [33] with some modification. In brief, it began with the initial capital letter of the genus, followed by the first two lowercase letters of the specific epithet. Subsequently, an underscore and the capital letters of the first two amino acids are displayed, followed by a numerical representation indicating the total count of amino acids in the sequence. When multiple sequences share identical names, a hyphen followed by a numerical identifier indicating its repetition was added.

### 2.7. 3D Modelling of Cathelicidin Domain and AMPs

The 3D structures of bat cathelicidin domain and peptides were modelled using the bioinformatics tool AlphaFold2 Colab Fold v1.5.5 with MMseqs2 [34]. The sequences provided in FASTA format were utilized to generate a PDB file, and the resulting structures were analysed and visualized with UCSF ChimeraX software [35].

### 2.8. Modelling the Cathelicidins and Flanking Genes

To visualize the genomic organization of cathelicidins and their flanking genes, the authors utilized the gggenes and ggplot2 packages in R software. The authors calculated the intra-exon distances in base pairs (bp) and determined the length of each sequence [36].

## 3. Results

### 3.1. Genome Mining

The search of the NCBI database revealed 41 species across 8 families within the order Chiroptera, yielding a total of 81 genomes, 62 of which were designated as Reference Sequences (Table S1). Genome mining was initiated using a probe to perform BLAST analysis on these genomes. After analysing scaffolds, contigs and chromosomes, the authors summarized the numbers of complete cathelicidins, incomplete sequences, nonfunctional cathelicidins and HDPs, as shown in Table S1. The results showed that only 12 species contained more than one cathelicidin gene.

The authors mapped the UTRs, exon sequences and strand orientations of all identified cathelicidin genes, leading to the discovery of 72 sequences. Notably, 44 of these sequences were identified for the first time, indicating that they were previously unannotated in the NCBI database. Exon 1 exhibited variable sizes, while exons 2 and 3 were conserved at 108 bp and 72 bp, respectively. Exon 4 displayed the greatest variability, reflecting the diversity of the HDPs (Table S2). The amino acid sequences were manually annotated and aligned, emphasizing the four characteristic cysteines and the sequences encoded by each exon. Finally, using NCBI's Conserved Domain Search Tool, the authors highlighted two distinct domains: the cathelin domain (pfam00666) [37], spanning from exon 1 to the initial amino acids of exon 4, and, in certain peptide sequences, the CAP18 domain [38] (Table 2).

**Figure 1:**
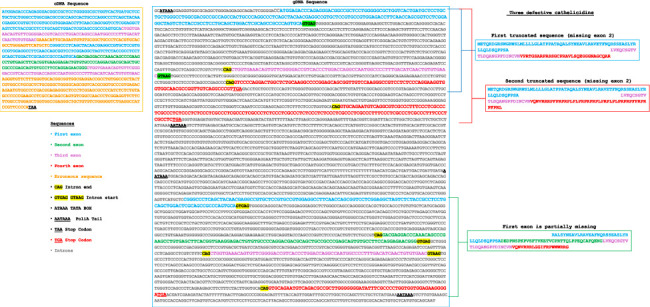
*Pteronotus mesoamericanus* (Mormoopidae) contains three nonfunctional cathelicidins. This figure illustrates the cDNA sequence obtained using the bioinformatics tool FGENESH following the mining process. The exon sequences are colour-coded as follows: exon 1 is represented in blue, exon 2 in green and exon 3 in purple. Erroneous cDNA sequences identified by the bioinformatics tool are highlighted in orange. Using a word processor, the authors identified the exon sequences within the gDNA. Additionally, our mining method revealed three sequences corresponding to exon 4, highlighted in red. The translation of these sequences may lead to the production of three nonfunctional cathelicidins. GenBank accession number: NW_026555320.1, isolate MK-2021, scaffold 533, whole genome shotgun sequence (gDNA: Start: 3,611,896–End: 3,617,153 bp).

Certain identified cathelicidins exhibit altered sequences, either due to incomplete or absent exons. For example, the authors found six complete and three defective cathelicidins in the genome of *Pteronotus mesoamericanus* (Mormoopidae). The manual analysis revealed an exon 1, followed by an exon 3 and two putative exons 4, each containing its respective stop codon. The absence of the second exon, combined with the fusion of the first and third exons with either of the two-fourth exons, could result in the production of two nonfunctional cathelicidins (Figure 1).

Further downstream in this genomic region, the authors identified a third incomplete cathelicidin, characterized by a missing segment at the beginning of exon 1, suggesting that it is also likely nonfunctional (Figure 1).

In other instances, such as in the genome of *Eonycteris spelaea*, a single nonfunctional cathelicidin was encoded due to the presence of a premature stop codon in the first exon (Figure S1).

Lastly, seven incomplete cDNA sequences from the NCBI database, representing three bat species (*Myotis myotis*, *M. davidii* and *Trachops cirrhosus*), showed short or N-filled scaffolds. However, six of these sequences included the exon 4, enabling the identification of the active peptide sequence (see Tables S1 and S3).

### 3.2. Alignment and Structural Analysis of Bat Cathelicidin Domains

**Figure 2:**
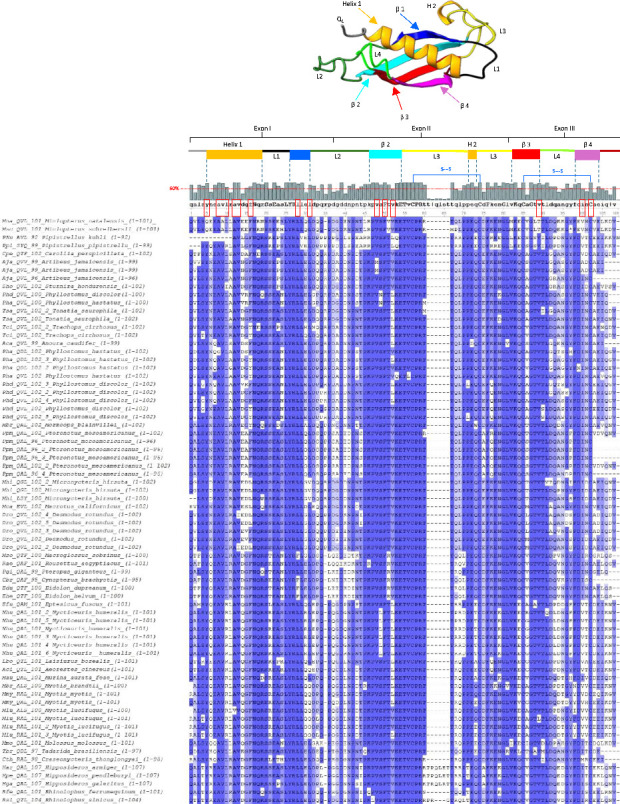
The alignment of 72 bat cathelicidin domains resulted in a per cent identity diagram, with higher values depicted in more intense blue. At the top, a consensus sequence and a histogram are presented. Each column above an amino acid indicates the percentage of the 72 sequences that have the same amino acid at that position (dominant amino acid). Capital letters indicate 100% identity, ‘+' indicates at least two dominant amino acid residues, and ‘−' indicates a dominant gap. The 3D structure derived from the consensus sequence comprises an α-helix, 4 loops and 4 β-strands, each highlighted in a characteristic colour with corresponding bars above the consensus sequence and is indicated using the following code: (H) Helix, (β) Beta strand, (L) loop. The disulphide bridges between the four cysteines are indicated in the consensus sequence. The 15-consensus sequence amino acids highlighted in the red rectangle represent the side chains responsible for establishing the van der Waals interactions. The regions encoded by each exon are indicated. The length of each analysed bat cathelicidin domain is specified next to the respective species name. The amino acid sequence alignment of the 72 cathelicidin domains was conducted using ClustalW with UGENE software. The 3D structure was generated using AlphaFold2 Colab Fold v1.5.5 with MMseqs2.

In the alignment of 72 bat cathelicidin domains, most positions were conserved, as indicated by the intense blue highlighting (Figure 2). The accompanying histogram revealed that 82 of the 102 positions in the consensus sequence exhibited a percentage identity of 60% or greater. However, only 19 amino acid residues in the consensus sequence were represented in uppercase, denoting their presence in 100% of the sequences analysed. Notably, four of these amino acids are cysteines that form disulphide bonds, which stabilize the β-sheet structure of the bat cathelicidin domain (pfam00666). The segment between positions 60 and 67 exhibited reduced sequence homology and contained an additional amino acid sequence within the second exon, which is characteristic of a limited number of bat species. The exclusion of these additional amino acids allowed us to create a three-dimensional structure to elucidate the most conserved positions within each secondary structure of this domain (Figure 2).

A comparative analysis of the cathelicidin protegrin-3 motif from *Sus scrofa* and the consensus sequence of bat cathelicidin domains was conducted to evaluate their folding patterns, based on X-ray diffraction studies by Sanchez et al. [39], which confirmed that protegrin-3 adopts a fold homologous to the cystatin family.

**Figure 3:**
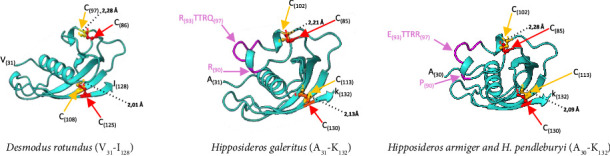
Depiction of segments of three cathelicidins from *Desmodus rotundus* (V_31_–I_128_), *Hipposideros galeritus* (A_31_–K_132_) and the two homologues from *H. armiger* and *H. pendleburyi* (A_30_–K_132_). In *D. rotundus*, the four cysteines (highlighted in orange and red) form disulphide bridges between positions 86–97 and 108–125, with distances of 2.28 Å and 2.01 Å, respectively. Despite an additional sequence insertion within this exon (highlighted in purple), the cysteines of the three species of *Hipposideros* genus (also highlighted in yellow and red) remain paired, capable of forming disulphide bridges, with similar distances of 2.21–2.28 Å and 2.09–2.13 Å for positions 85–102 and 113–130, respectively. The figures have been computed using ColabFold v1.5.5: AlphaFold2, MMseqs2 and USCF ChimeraX version 1.4 (2022-06-03).

The three-dimensional structure of the bat cathelicidin consensus sequence featured an α-helix, and four antiparallel β-strands linked by loops, forming a twisted β-sheet stabilized by disulphide bridges, between cysteine residues at positions 57–74 and 85–102 (Figure 2). All 15 amino acids identified by Sanchez et al. [39] as critical for van der Waals interactions in the cystatin-like domain were present in the bat consensus sequence. However, leucine at position 45 of the α-helix was conserved in only six bat species, while phenylalanine, another hydrophobic amino acid, was more common in the remaining species. Figure 2 illustrates these 15 amino acids, enclosed within a red rectangle and showing nearly 100% identity, as indicated by the deep blue highlights. To verify the importance of these 15 amino acids for proper domain folding, the authors analysed the van der Waals interactions within the domain following the substitution of each residue with glycine. Comparison of the resulting structures revealed alterations in these interactions and, ultimately, a disruption of the beta‐sheet's correct folding (Figure S3). As previously noted, the authors identified cathelicidins with varying exon sizes. Within the Hipposideridae family, *Hipposideros armiger* and *H. pendleburyi* produce identical cathelicidins, while *H. galeritus* synthesizes a homologous sequence, all featuring a six-amino-acid extension in the second exon. Although these sequences differ, their positions within the protein remain consistent, with R_(90)_-R_(93)_TTRQ_(97)_ in *H. galeritus* and P_(90)_-E_(93)_TTRR_(97)_ in *H. armiger* and *H. pendleburyi*. These amino acids alter the distances between the four cysteines characteristic of cathelicidin proteins (see Table 2). When comparing the cathelicidin of the *Hipposideros* genus with that of *Desmodus rotundus*, which lacks the additional extension, it was observed that despite the six extra amino acids in *Hipposideros*, the distances between the disulphide bridges remained similar (the typical range for disulphide bridge lengths is between 2.05 Å and 3 Å [40]). Furthermore, the amino acids establishing van der Waals contacts seemed to maintain these interactions. This indicates that the extension did not alter the protein's three-dimensional structure (Figure 3). This suggests that the extension in the second exon could be a distinctive feature of the *Hipposideros* genus and that these extra-length sequences do not disrupt the proper folding of the β-sheet.

*Rhinolophus sinicus* (Rhinolophidae) possesses a three-amino-acid extension within the second exon (Table 2). Similar to the Hipposideridae family, this extra-length sequence did not hinder the formation of the disulphide bridge between cysteines 85–99 and van der Waals contacts (Figure not shown). Since this extension was unique to *R. sinicus* and absent in *R. ferrumequinum*, the authors cannot definitively attribute it as distinct feature of this genus. Additionally, the lack of genomic data from other individuals of this species prevents us from determining whether this sequence is a species-specific trait or an exclusive mutation of the sequenced specimen.

**Figure 4:**
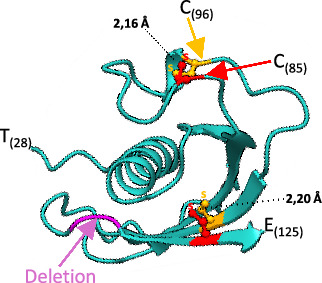
The three-dimensional structure of the segment (T28-E125) of cathelicidin from *Miniopterus schreibersii* featured a deletion of a single amino acid between positions 118 and 119. Despite the reduced sequence length between cysteines 85 and 96, there appeared to be no impact on disulphide bridge formation or the overall protein folding.

Furthermore, *Miniopterus schreibersii* (Vespertilionidae) exhibited length variations due to the deletion of one amino acid between positions 118 and 119 (Table 2, indicated by a red asterisk in the cathelicidin sequence). This deletion occurred within another loop region, resulting in a distance of 9 amino acids instead of 10 between the two cysteines in the third exon, which likely did not impede disulphide bridge formation (Figure 4).

Finally, considering the key regions of the conserved domain, a recombinant cathelicidin can be designed based on the consensus sequence identified in the per cent identity diagram (Figure 2) and incorporating a consensus signal peptide sequence. Furthermore, inclusion of one of the peptide types identified in this study may offer practical applications in future research (Figure S4).

### 3.3. Phylogenetic Analysis of Bat Cathelicidin Domain

**Figure 5:**
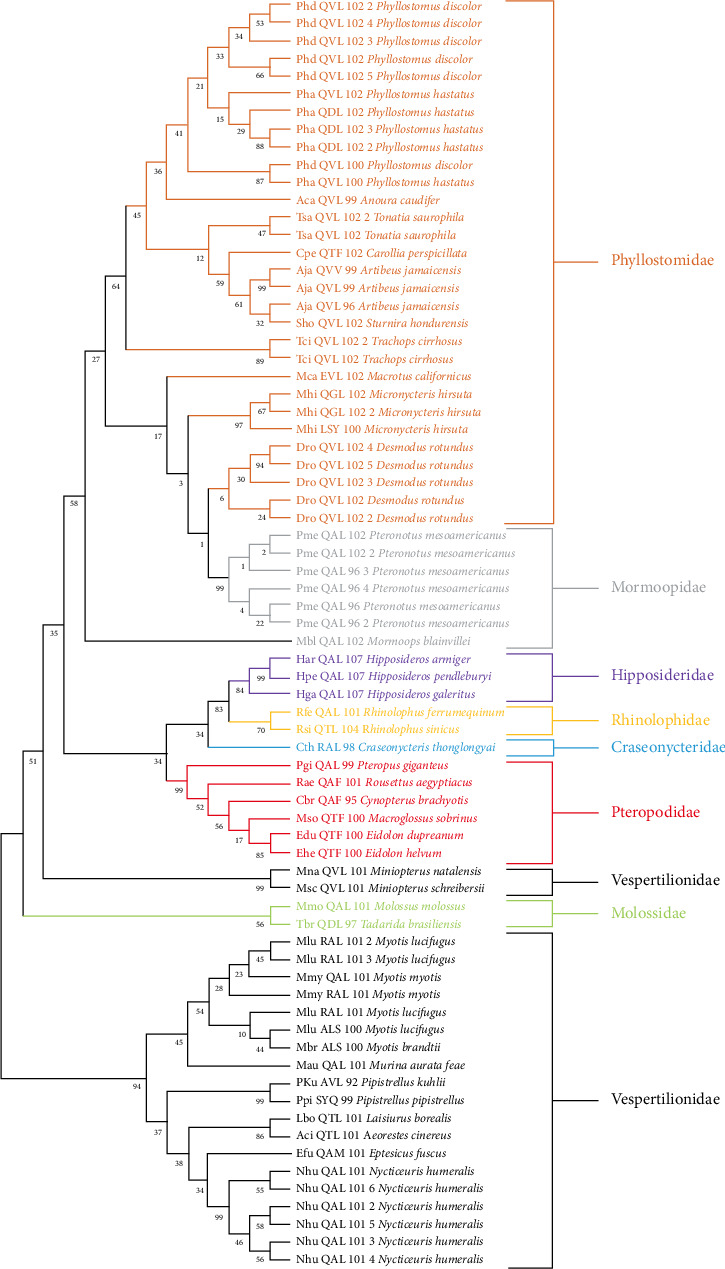
Phylogenetic tree derived from the conserved cathelicidin domain in bats, constructed to evaluate the degree of conservation across the eight Chiroptera families examined and its potential applicability as a phylogenetic marker. The resulting tree reveals clustering of species according to their taxonomic order. Notably, the genera Miniopterus (Vespertilionidae) and Pteronotus (Mormoopidae) deviate from the main clusters of their respective families, consistent with previously reported taxonomic divergences for these taxa, and supporting the potential use of this conserved domain as a phylogenetic marker. For tree construction, the 72 conserved domains identified were aligned using MUSCLE, and the phylogeny was inferred using the maximum likelihood method, applying the Jones–Taylor–Thornton (JTT) model of amino acid substitution with gamma distribution and invariant sites (JTT + G + I). Node support for the unrooted tree was assessed by bootstrap analysis with 1000 replicates [30].

The aim of this analysis was to evaluate the conservation of the bat cathelicidin domain across the eight families studied and to assess its potential as a phylogenetic tool. The phylogenetic tree (Figure 5) generally showed the clustering of species according to their taxonomic order. The Vespertilionidae and Molossidae families appear to have the most ancestral cathelicidins, while the tree also revealed a close relationship among the sequences of the Pteropodidae, Craseonycteridae, Rhinolophidae and Hipposideridae families. More recent cathelicidins seem to have emerged in the Mormoopidae and Phyllostomidae families. However, cathelicidins in the genus *Miniopterus* of the Vespertilionidae family diverged earlier than those in other species of this family, consistent with the taxonomic divergences observed in the classification of this genus [41]. Another controversial branching was noted within the Mormoopidae family: the sequences of *Pteronotus mesoamericanus* appeared to align with the Phyllostomidae family, while *Mormoops blainvillei* resembled an independent lineage. This finding aligns with the earlier classification of Mormoopidae within Phyllostomidae [42].

### 3.4. Introns

**Figure 6:**
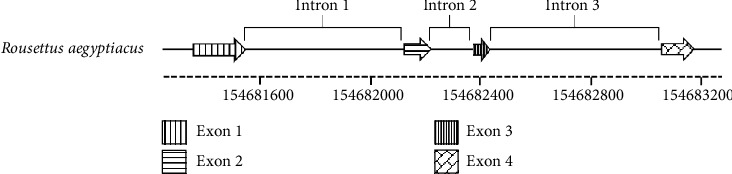
Example of a bat cathelicidin gene: The genomic structure of a cathelicidin from Rousettus aegyptiacus shows four exons and three introns. The arrows indicate its position on the forward strand of the gDNA molecule (NCBI NW_023416307.1). Sequence lengths are expressed in base pairs (bp). Visualization was performed using the ‘gggenes' and ‘ggplot2' packages in R [36].

For the mining process, it was necessary to conduct a delineation of the intron sequence in all the genomic DNAs (gDNAs) analysed. As depicted in Figure 6, the cathelicidin gene was divided into three introns and four exons. The precise genomic locations in each studied species, expressed in base pairs (bp), are provided in Table S2.

During our intron study, the authors identified highly conserved initiation and termination sequences critical for the mining method to determine exon locations. These sequences included the 5′ and 3′ intron splice sites: GT and AG, respectively [43] (Table 3). In many instances, introns began with the sequence (GTGAG), while in some species, it started with GTAAG. Each intron was generally terminated with the triplet (CAG); however, in other species, it ended with TAG (Table 3). These sequences aligned with the predominant splicing consensus nucleotides found in mammals [44].

On the one hand, the introns length was variable. The size of intron 1, for example, was typically 603 bp, with the length occasionally falling below 270 bp or exceeding 1200 bp. Intron 2, which separates exons containing a substantial portion of the conserved domain, was the shortest, with a length of around 150 bp. Finally, the length of the intron 3 varied considerably, ranging from 500 to 600 bp (Table 3).

The cathelicidin amino acid sequences of *Hipposideros armiger* and *Hipposideros pendleburyi* were identical. However, their intron sequences differ. In the alignment of the two 646-bp intron 1 sequences, the authors found a 99.23% identity, with only 5 base pairs differing (Figure S2.A). The intron 2 varied in length with *H. armiger* having 134 bp compared to 137 bp in *H. pendleburyi*, resulting in 97.08% identity (Figure S2.B). A similar variation was observed in the intron 3, with lengths of 578 bp for *H. armiger* and 574 bp for *H. pendleburyi*, giving an identity of 98.44% (Figure S2.C). Although these species share the same cathelicidin, the intron sequences exhibited slight differences in length and sequence.

### 3.5. Genomic Organization

**Figure 7:**
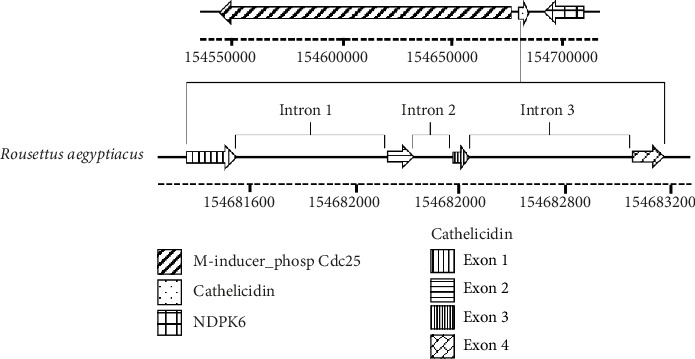
Illustration of the cathelicidin gene genomic localization in relation to its flanking genes. Remarkably, the orientation of cathelicidin gene is consistently opposite to that of the flanking genes, irrespective of whether it is located on the forward or reverse strand. The sequence length is expressed in base pairs (bp). Visualization was conducted using the ‘gggenes' and ‘ggplot2' functions from the R package [36].

In the context of genomic organization, our investigation also involved identifying flanking genes associated with cathelicidins. In most species, the authors observed an upstream gene encoding an enzyme from the nucleoside diphosphate kinase family (NDPk). Additionally, the authors identified a downstream gene belonging to the mitosis-inducing phosphatase protein family (Cdc25) (Figure 7).

It is important to emphasize that the authors found various cathelicidin genes in 10 of the 41 species under investigation, all of them appear consecutively and are located between these flanking genes, forming a genetic cluster (Table S2).

However, in this research, only four species have their genomes completely sequenced, allowing us to locate the cathelicidin gene at the chromosomal level. In *Desmodus rotundus* (Phyllostomidae), the cathelicidin gene was located on chromosome 8; in *Phyllostomus discolor* (Phyllostomidae) and *Eidolon dupreanum* (Pteropodidae), it was on chromosome 7; and in *Rhinolophus ferrumequinum* (Rhinolophidae), it was on chromosome 17 (Table S2). Therefore, the chromosome on which the cathelicidin cluster is located does not appear to be highly conserved throughout the order Chiroptera.

### 3.6. Peptides

In our final analysis, the authors provided a detailed description of the hypervariable domain encoded by the fourth exon. By performing genome analysis of Chiroptera using our mining method and considering the cleavage by the enzyme elastase at the first valine in exon 4 [32], the authors identified 78 peptides: 69 from complete cathelicidin sequences, 5 from incomplete sequences and 4 from nonfunctional sequences (Tables S1 and S3). Additionally, using the NCBI Conserved Domain bioinformatics tool, the authors identified 22 peptides containing the CAP18 domain, as highlighted in red in Table 4. Peptide sequences were manually aligned, focussing specifically on amino acids within the CAP18 domain (isoleucine, leucine, phenylalanine and valine). This approach also allowed us to identify an additional 23 sequences with significant similarity to the CAP18 domain, which were highlighted in purple. Notably, the conserved domain of the cathelicidin extended into exon 4 by a few amino acids, aiding in peptide identification. Finally, the authors provided a summary of the three-dimensional structures of the peptides, modelled using the AlphaFold bioinformatics tool (Table 4).

The authors followed the structural classification of HDPs by Koehbach and Craik [45] to categorize the predicted bat cathelicidin peptides into eight types, based on features such as the presence or absence of the CAP18 domain, proline-rich or arginine-rich sequences, and their three-dimensional structures (Table 4). The most prevalent structure observed in the predicted bat cathelicidin peptides was the α-helix, which predominates in both Type 1 and Type 3 peptides. Type 1 peptides contained the CAP18 domain, whereas Type 3 peptides did not. Peptides classified as Type 2 (containing the CAP18 domain) and Type 5 (lacking the CAP18 domain) featured two α-helices connected by a β-turn (Table 4). In contrast, Type 4 peptides exhibited an α-helix associated with a β-sheet.

*Pteronotus mesoamericanus*, *Phyllostomus discolor* and *P. hastatus* are known to biosynthesize peptides characterized by proline- and arginine-rich sequences. In these peptides, proline residues reduce structural stability due to the absence of free hydrogen atoms, which prevents hydrogen bond formation and results in an extended structure [45], classified as Type 6. Additionally, the authors identified another category of extended structures, distinguished by high arginine content but lacking proline, termed Type 7 (Table 4).

Finally, *Pteronotus mesoamericanus* showed a distinctive predicted peptide (Pme_PR31) with an extended structure, characterized by the presence of two cysteines separated by a sequence of 14 amino acids. Pme_PR31 could form a disulphide bridge between these two cysteine residues or possibly between two peptides, resulting in the formation of a dipeptide. It was classified as a Type 8 peptide (Table 4).

## 4. Discussion

The main objective of this study was to identify all the cathelicidins and its AMPs across 41 bat species available in the NCBI database. The bat cathelicidin conserved domain facilitated the precise localization of the first three exons of the cathelicidin gene within gDNA. However, due to variations in the length of exon 4, accurate identification using bioinformatics tools proved challenging, often resulting in incorrect peptide predictions, even when the initial amino acids were part of the conserved domain. Similar challenges were observed with the identification of 5′ UTRs and PolyA tails, which were frequently misidentified. Furthermore, variability in the positioning of specific splicing sequences within the gDNA introduced additional complications [46]. To overcome these challenges, the authors employed a combined bioinformatics approach, supplemented by manual curation, to accurately determine and annotate the full-length sequences of each cathelicidin.

Using this method, the authors identified cathelicidin genes in *Anoura caudifer* (Phyllostomidae) and in species from the family Pteropodidae, in contrast to the findings of Castellanos et al. [23], who reported the absence of cathelicidins in these species.

In our mining method, intron sequences were crucial for accurately identifying exon positions. This approach allowed us to detect defective cathelicidin genes with exon deletions, incomplete exon sequences or erroneous stop codons within exons. For example, analysis of *Phyllostomus hastatus* gDNA using the bioinformatics tool FGENESH revealed that the third identified cathelicidin gene lacked the second exon. Corvelo et al. [46] emphasize the importance of accurately identifying the 3′ splice site for computational methods to predict intron sequences. However, manual analysis showed the 3′ splice site adjacent to the missing exon, with the terminal triplet AGG. Typically, spliceosome activity targets the consensus sequence AG at the 3′ splice site, but this cathelicidin exhibited 2 G bases at this position. This suggests a potential dysfunction of the cathelicidin gene arising from alterations in the conserved nucleotides of the splicing sequence [47]. While the bioinformatics tool did not detect this anomaly, the genomic DNA contained the complete exon sequence adjacent to the erroneous splicing site.

As previously mentioned, analysis using the NCBI Conserved Domain Database identified a conserved cathelin domain (pfam00666) in bat cathelicidin sequences. Alignment of bat conserved domains revealed a consensus sequence with low variability at each amino acid position, indicating that, unlike birds [30] or other mammals such as pigs (*Sus scrofa*) [39], bats possess a unique type of cathelicidin. One of the cathelicidins in *Sus scrofa*, protegrin 3, shared numerous amino acids with the cystatin-like domain. Our studies confirmed that the three-dimensional structures of the conserved domain of protegrin-3, modelled by Sanchez et al. [39] using X-ray diffraction, and the consensus sequence of the conserved domain in bat cathelicidins were nearly identical. Therefore, the conserved domain of bat cathelicidins adopts a cystatin-like structure.

The alignment of the conserved domain highlighted similarities among bat cathelicidins, while their variability enabled phylogenetic analysis. Cathelicidins cluster in the phylogenetic tree according to taxonomic order, suggesting their sequences could serve as reliable taxonomic markers. However, the phylogenetic tree revealed controversial branching within the Mormoopidae family. Specifically, *Pteronotus mesoamericanus* was positioned in line with its early 20^th^-century classification within Phyllostomidae. Historically classified as a subfamily (Chilonycterinae) until 1972, Mormoopidae was subsequently elevated to family status [42]. Pavan and Marroig [42] conducted a taxonomic revision using molecular markers, morphology and ecology, reaffirming Mormoopidae as an independent family while emphasizing the complexity of the genus *Pteronotus*. Given this complexity, the conserved cathelicidin domain in *P. mesoamericanus* supports its classification within Phyllostomidae. Therefore, an integrative study that considers multiple factors, including this conserved domain, is essential for accurate phylogenetic analysis.

A similar pattern was observed in the genus *Miniopterus* within the Vespertilionidae family, which appears as an independent family. These findings align with genetic and taxonomic studies by Miller-Butterworth et al. [41], which proposed that the taxonomic position of *Miniopterus* remains unresolved. Factors such as morphology, embryology, immunology and recent genetic analyses have cast doubt on this classification. Their studies confirm the uniqueness of *Miniopterus* and support the proposal to elevate these bats to full familial status, consistent with our phylogenetic tree.

In addition, the initial and terminal intron sequences of the *Pteronotus* and *Miniopterus* genera exhibit notable differences and could serve as potential phylogenetic markers to be accounted.

An example related with introns as phylogenetic markers in bat was observed after identifying two species synthesizing the same cathelicidin. Comparative analysis of their intron sequences revealed slight differences in both sequence composition and length, suggesting that cathelicidins introns could be an effective strategy for distinguishing closely related species. However, to draw definitive conclusions from the phylogenetic analysis and intron sequences, further genomic analyses of specimens and species from the Chiroptera order are essential.

Interestingly, when comparing this phylogenetic tree with those of other vertebrate groups that have multiple cathelicidin types, such as birds, amphibius or even mammals [30, 39, 48], the pattern observed in bats appeared simpler. Unlike birds, where species often formed distinct clusters corresponding to different cathelicidin types [30], bat species with multiple cathelicidin genes did not display such clustering. Therefore, all cathelicidins belong to the same type, having derived from a common gene. This conclusion is further supported by the identity graph, where the consensus sequence of the conserved domain shows over 70% identity in the majority of positions.

Regarding genomic organization, the chromosomic location of the cathelicidin genes is variable and can differ even between species of the same family. Nonetheless, the authors identified the bat cathelicidin gene in a context similar to that of *Homo sapiens* and *Mus musculus*, flanked by the Cdc25 (mitosis-inducing phosphatase), known for its regulatory role in eukaryotic mitosis and control of cellular division processes [49] and NDPk (nucleoside diphosphate kinase) gene families. This kinase is crucial for generating nucleoside triphosphates, essential for DNA and RNA synthesis, cell division, macromolecular metabolism and overall growth [50]. During the mining process, identifying these flanking genes could help pinpoint the locations of cathelicidins within the Chiroptera order and potentially in other mammals (Figure 7). It is important to highlight that most of the bats studied possess a single cathelicidin gene; however, a few species exhibit multiple genes, with differences located within the exon 4, which encodes the active peptide [22, 23].

The fourth exon encodes the active site of cathelicidins, with activation occurring when the elastase enzyme cleaves the first valine at the start of this sequence [51, 52]. Initially, the authors relied exclusively on bioinformatics tools, which faced several challenges, particularly in accurately predicting peptide sequences from gDNA. However, our mining approach led to the identification of 78 peptides, 25 of which match sequences available in the NCBI database, demonstrating the high accuracy of our method.

In the next step, the authors employed bioinformatics methods to align the peptides, which revealed scattered clustering likely due to their varying lengths, while others exhibited alignment tendencies primarily based on their initial or terminal amino acids. To address this, the authors focussed on specific amino acids within the peptides' hydrophobic regions, performing a manual alignment centred on these amino acids and conserved domains or similar sequences. This region might be crucial for peptide functionality, given its high conservation across the analysed peptides. The precision of this alignment was essential for classifying the peptides into eight groups based on their structural characteristics and sequences (Table 4). The most prevalent structure observed was the α-helix, followed by structures with two α-helices connected by a β-turn. Notably, peptides with an α-helix associated with a β-sheet were identified, resembling the insect defensin CSαβ defensin formalin (1/CA). Additionally, extended structures were found, characterized by high proline and arginine content that prevents hydrogen bond formation. The authors also identified a peptide with two cysteine residues separated by a sequence of 14 amino acids. This configuration could allow for a disulphide bridge between these residues or the formation of two intermolecular disulphide bridges between two peptides, potentially resulting in a dipeptide. This structure may be similar to peptides synthesized by *Sus scrofa* (Protegrin 1–5), known for their two disulphide bridges [45, 53].

Regarding the peptide sequences, the bioinformatics tool Conserved Domain identified the presence of the CAP18 domain in many cases. This domain was originally characterized as a lipopolysaccharide-binding protein in rabbit granulocytes, with a propensity to adopt an α-helical conformation [38]. Notably, some peptides contained a partial sequence of this domain and were successfully identified using our manual alignment approach, whereas the NCBI bioinformatics tool failed to detect them. In conclusion, the authors propose a peptide classification system that may facilitate future studies on structure–activity relationships and mechanisms of action.

## 5. Conclusions

This study underscores the need to combine bioinformatics-based mining with manual analysis of gDNA sequences for accurate cathelicidin annotation. Bat cathelicidins exhibit a conserved domain similar to pfam00666, with a β-sheet structure identical to the cystatin-like domain. The low structural and sequence variability of this domain suggests the presence of a single cathelicidin type in bats. Analysing intron splicing sequences together with conserved domains could be an effective approach for accurately identifying cathelicidin genes. However, additional genomic studies across Chiroptera are necessary. While the number and location of cathelicidin genes in bat genomes vary, they are consistently flanked by the same genes. Despite high variability in peptides encoded by exon 4, eight distinct types were identified based on sequence and structural features. In summary, the 41 species studied share a unique cathelicidin type capable of producing eight distinct types of peptides.

## Figures and Tables

**Table 1:** (A) Cathelicidins obtained from the NCBI protein database. (B) Consensus sequence obtained by combining the cathelicidins from the mentioned four bat species.

	Sequences
(A)	>XP_036905727.1 cathelicidin antimicrobial peptide isoform X1 [*Sturnira hondurensis*]
	>XP_024421797.1 cathelicidin antimicrobial peptide isoform X1 [*Desmodus rotundus*]
	>XP_024421797.2 cathelicidin antimicrobial peptide [*Desmodus rotundus*]
	>XP_036905728.1 cathelicidin antimicrobial peptide isoform X2 [*Sturnira hondurensis*]

(B)	>Consensus sequence (Probe)/1–164
	METQKDSLLPGCWPLLLLLLGLAVPPATAQVLSYNEAVIAAIDDFNQRSSEASLYRLLELDQQPPDGDDNPDTPKPVSFTVKETVCPRTTQLPPEQCEFKENGLVKQCAGTVTLAQANDSFDIDCADIPDVGIRSGVQRIVDKIRDIGRRINDFFSNLFPRGVS

**Table 2:** Amino acid sequences of bat cathelicidin, which are composed of four exons. The exons are colour-coded as follows: the first exon in red, the second exon in blue, the third exon in green and the fourth exon in black. The table also highlights specific structural and functional features within these sequences: Conserved cathelin domain: This domain is a conserved feature among these proteins, spanning from the first exon through to the initial amino acids of the fourth exon. It is indicated by underlined letters. Conserved cysteines: These cysteines are highlighted in yellow. The intervals between these cysteines are consistent, with distances of 10, 10 and 16 amino acids, respectively. Notably, some species have an additional sequence (in purple) between the first two cysteines. CAP18 domain: The CAP18 domain is present within the fourth exon in certain species and is indicated by letters in dark red. Novel sequences: Of the 72 proteins listed, 44 are reported for the first time and do not have a reference number. These novel sequences are highlighted in orange within the sequence number box. Finally, the species *Hipposideros armiger* and *H. pendleburyi* synthetize the same cathelicidin; for that reason, the authors have annotated 72 proteins and 71 different sequences. A red asterisk indicates an amino acid deletion in the third exon of *Miniopterus schreibersii* (Vespertilionidae). The amino acids highlighted in blue within the first exon occur less frequently at these specific positions.

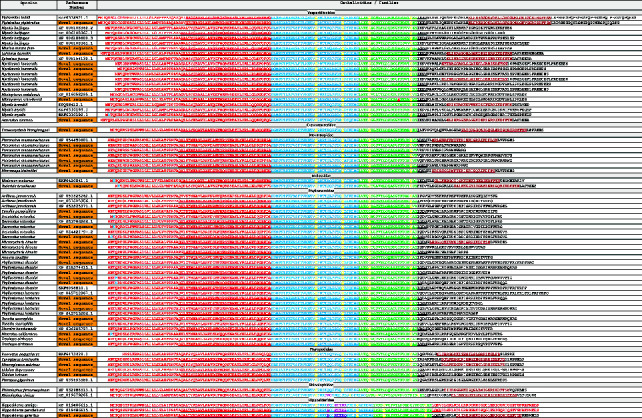

**Table 3:** Intron sizes for the 72 cathelicidins found in the eight families of the order *Chiroptera*, along with the five initial bases and 3 terminal bases of each intron. These sequences include the 5′ and 3′ consensus splice sites highlighted as GT and AG, respectively. The most prevalent sequences are GTGAG for the start and CAG for the end. Cathelicidin genes with a different sequence are highlighted in blue, orange or green. The third cathelicidin of *Phyllostomus hastatus* shows an anomalous base pair in the 3′ splice site, highlighted in yellow.

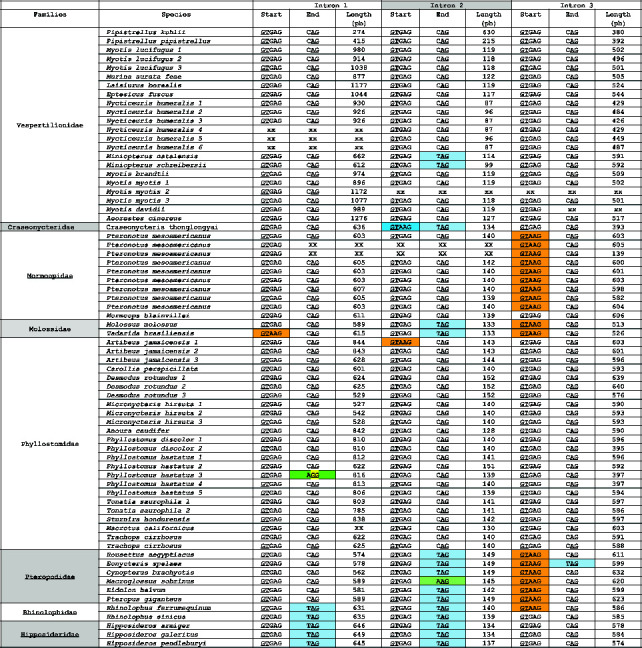

**Table 4:** Alignment of 78 predicted peptides from bat cathelicidins. The 22 sequences containing the CAP18 domain are highlighted in red, while the 23 sequences bearing a similarity to this domain are highlighted in purple. Amino acids, isoleucine (I), leucine (L), proline (P) and valine (V), crucial for the alignment process, are highlighted in different colours. Peptide names follow the format outlined in the Materials and Methods (Section 2.6). Each of the eight peptide types is highlighted in a specific colour, with its characteristic 3D structure shown alongside the sequence type.

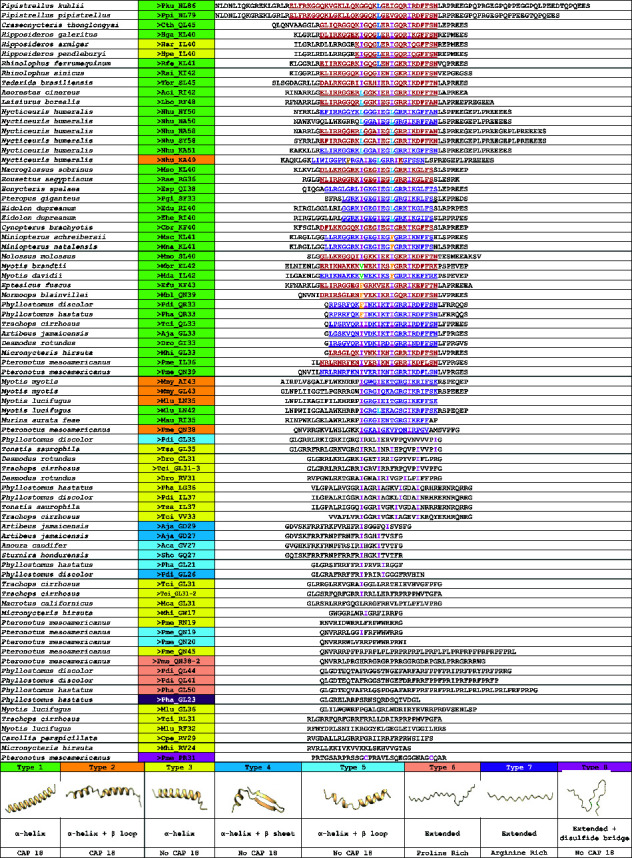

## Data Availability

The data that support the findings of this study are available upon request from the corresponding author.

## Supporting Information

Supporting Information 1 Figure S1: Fragment of the gDNA (1821556 pb-1824372pb) of Eonycteris spelaea encodes a single nonfunctional cathelicidin.

Supporting Information 2 Figure S2.A: This figure shows the alignment of the first intron in *Hipposideros armiger* and *H. pendleburyi*. Figure S2.B: This figure shows the alignment of the second intron in *Hipposideros armiger* and *H. pendleburyi*. Figure S2.C: This figure shows the alignment of the third intron in *Hipposideros armiger* and *H. pendleburyi*.

Supporting Information 3 Figure S3: This figure shows the Van der Waals interaction analysis of the conserved and modified domains.

Supporting Information 4 Figure S4: Representation of potential recombinant bat cathelicidins.

Supporting Information 5 Table S1: Summary of the cathelicidins, incomplete sequences, nonfunctional cathelicidins and antimicrobial peptides (AMPs) identified in the analysis of 62 NCBI Reference Sequences.

Supporting Information 6 Table S2: Summary of NCBI reference sequences of genomic DNA (gDNA) used for mining. It details the positions of cathelicidins' UTRs and exon sequences in base pairs (bp) from the start of the chromosome, contig or scaffold, along with sequence lengths.

Supporting Information 7 Table S3: Incomplete cathelicidin sequences from three bat species displayed scaffolds that were either incomplete or comprised of ambiguous ‘N' bases.
