# Supplementary material for: Genome Mining and Structural Study of Cathelicidins Across Chiroptera Species
Source: Biochem Res Int. 2025 Sep 23;2025:5461549. doi: 10.1155/bri/5461549 (PMC12483743; doi:10.1155/bri/5461549)
Supplement: Supporting Information 1 — Figure S1: Fragment of the gDNA (1821556 pb-1824372pb) of Eonycteris spelaea encodes a single nonfunctional cathelicidin. [file 5461549.f1.docx]

**Stop**

**Stop**

**First exon**

**Second exon**

**Third exon**

**Fourth exon**

**Figure S1.** Fragment of the gDNA (1821556 pb-1824372pb) of *Eonycteris spelaea* encodes a single cathelicidin that is non-functional due to a premature stop codon in the first exon. The exon sequences are color-coded as follows: the first exon in blue, the second exon in green, the third exon in purple, and the fourth exon in red. The stop codon within the first exon is in yellow. It is noteworthy that an erroneous sequence occurs 3 bp upstream of the stop codon. *Eonycteris spelaea* isolate SIG01 000144F, whole genome shotgun sequence (4778326 bp). Accession:.PUFA01000145 1. DNA linear.
