# Supplementary material for: Genome Mining and Structural Study of Cathelicidins Across Chiroptera Species
Source: Biochem Res Int. 2025 Sep 23;2025:5461549. doi: 10.1155/bri/5461549 (PMC12483743; doi:10.1155/bri/5461549)
Supplement: Supporting Information 2 — Figure S2.A: This figure shows the alignment of the first intron in Hipposideros armiger and H. pendleburyi. Figure S2.B: This figure shows the alignment of the second intron in Hipposideros armiger and H. pendleburyi. Figure S2.C: This figure shows the alignment of the third intron in Hipposideros armiger and H. pendleburyi. [file 5461549.f2.docx]

First intron

*Hipposideros armiger*

>armigerintron1 (646 pb)

GTGAGTTGGGGAAGGGGGCTGGGGAGGGAGACCCTGCTTCTATCAGGTGTGGCCATATTGCCACTCCTTTTGCTCATGCTGGACCGCTTGTCGGGAAGGCACTTCTCCCTTTAAGTGGGATCCTACCTCTTCCGGGAAACCTTCCCAGAGCTGTGTACCCTCCCAGCACAACAGCTTCCTGCCTTAGAATCACTACTGTATGGAGAGGTGACCCCCACACCCCCCCATTCAGGCTTCAGGGACTTCTGAGAGTTCCAGGGACAAGAATGGGGTCATTGGTTCTGGGATGTGACTTCCCTGCTTTAAGCCCCGTCTGCTCGCCATTGTCTCCCACGCAGGAAAGAGCTCTGCTCAGCCCTGAGGTTCCAGTGGTAATGTCTTTCCTCCAGGAGGGCCCCAATTTCCCTCAGCCTCCTTAGAGCTTGTCTGAGGTCCCCTCCTGCTCTCTGTATGCTTGGTGAGGGCAGGATGGGCTCTGTTCTCCTCACCTCTGTGCCTCAGCACCAAGCCCAGTGCCAGGCACACAGCAGGGGCTGTTGAAAAGCTGATTTCTCGGTGGTGTTGGGGGCGGGGGGAGATAGGGAGACAGATCAGAGAAGGCGAGCAGGAGCCCAAGCCCACTGTCCCTGCTTTCTCCCCTGGCTAG

*Hipposideros pendleburyi*

>pendleburyintron1 (646 pb)

GTGAGTTGGGGAAGGGGGCTGGGGAGGGAGACCCTGCTTCTATCAGGTGTGGCCATATTGCCACTCCTTTTGCTCATGCTGGACCTCTTGTTGGGAAGGCACTTCTCCCTTTAAGTGGGATCCTACCTCTTCCGGGAAACCTTCCCAGAGCTGTGTACCCTCCCAGCACAACAGCTTCCTGCCTTAGAATCACTACTGTATGGAGAGGTGACCCCCACACACCCCCATTCAGGCTTCAGGGACTTCTGAGAGTTCCAGGGACAAGAATGGGGTCATTGGTTCTGGGATGTGACTTCCCTGCTTTAAGCCCCGTCTGCTCGCCATTGTCTCCCACACAGGAAAGAACTCTGCTCAGCCCTGAGGTTCCAGTGGTAATGTCTTTCCTCCAGGAGGGCCCCAATTTCCCTCAGCCTCCTTAGAGCTTGTCTGAGGTCCCCTCCTGCTCTCTGTATGCTTGGTGAGGGCAGGATGGGCTCTGTTCTCCTCACCTCTGTGCCTCAGCACCAAGCCCAGTGCCAGGCACACAGCAGGGGCTGTTGAAAAGCTGATTTCTCGGTGGTGTTGGGGGCGGGGGGAGATAGGGAGACAGATCAGAGAAGGCGAGCAGGAGCCCAAGCCCACTGTCCCTGCTTTCTCCCCTGGCTAG

Alignment:


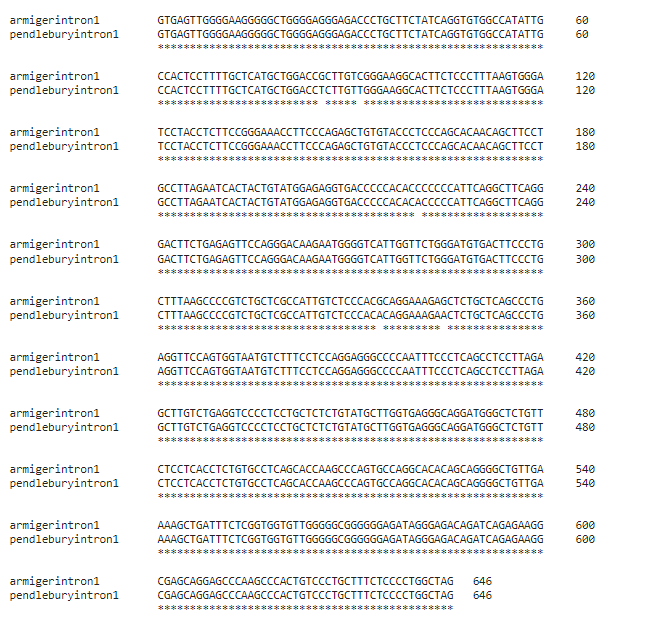

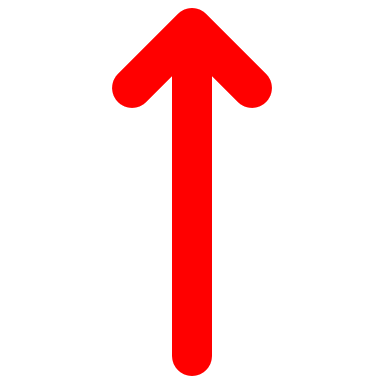

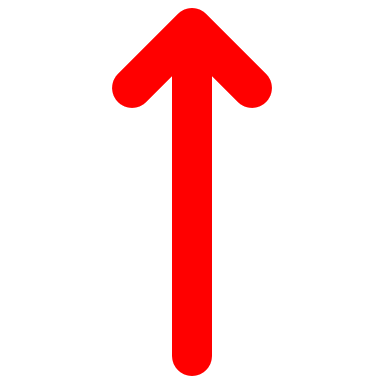

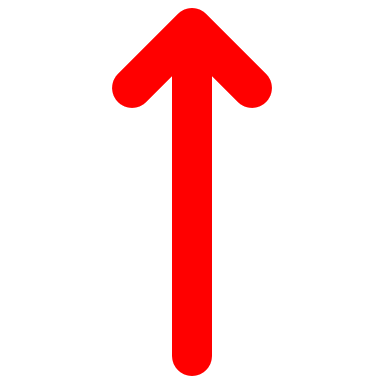

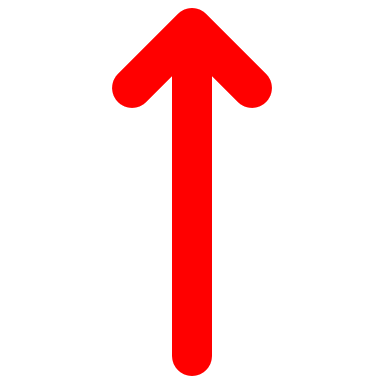

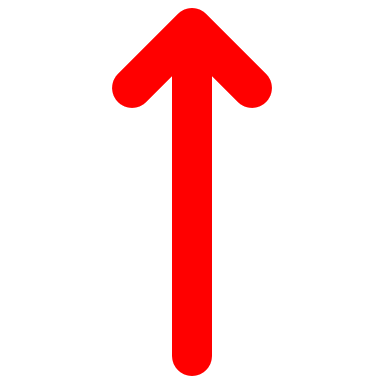


| Intron 1 | *Hipposideros armiger* | *Hipposideros pendleburyi* |
| --- | --- | --- |
| Length (bp) | 646 | 646 |
| Differences (bp) | 5 | |
| % Alignment | 99, 23% | |

**Figure S2.A.** The alignment of the first intron in *Hipposideros armiger* and *H. pendleburyi* reveals five nucleotide differences and an alignment percentage of 99.23%. (https://www.genome.jp/tools-bin/clustalw).

Second Intron

*Hipposideros armiger*

>armigerintron2 (134 pb)

GTGAGGCTGAGGACTAGGGTCTGGGGGTGCTGGCGGGTGCCTTCCCAGATGCTGAACAATTTCCAGACACTGGGGTGAGGTTGGGAGGTTGTGGTTCAGGGGTTCCAGTTTGACCTTGAGCCTCCCCTTTTTAG

*Hipposideros pendleburyi*

>pendleburyintron2 (137 pb)

GTGAGGCTGAGGACTAGGGTCTGGGGGTGCTGGCGGGTGCCTTCCCAGATGCTGAACAATTTCCAGACACTGGGGTGAGGTTGGGAGGTTGTTGTGGTTCAGGGGTTCCAGTTTGACCTTGAGCCTCCTCTTTTTAG

Alignment:


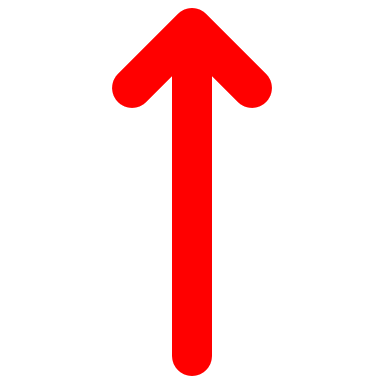

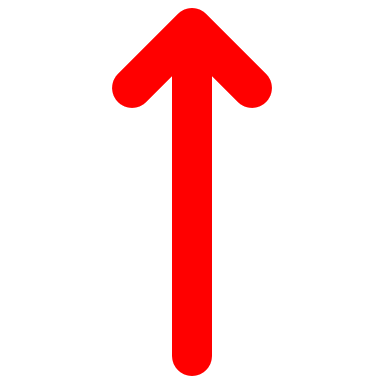

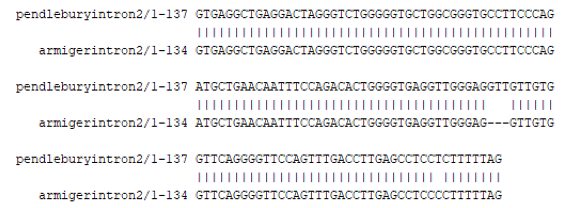

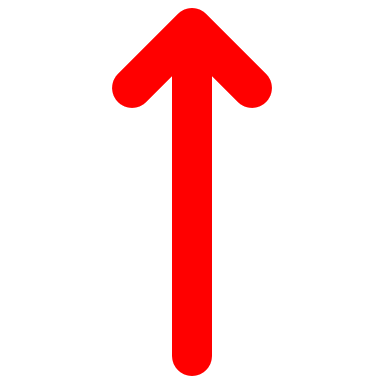

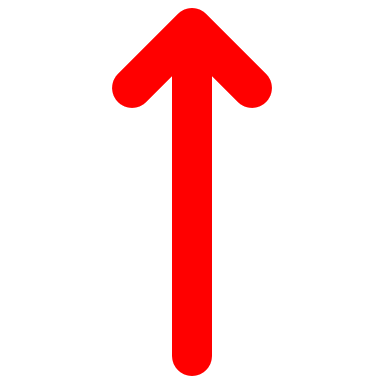


| Intron 2 | *Hipposideros armiger* | *Hipposideros pendleburyi* |
| --- | --- | --- |
| Length (bp) | 134 | 137 |
| Differences (bp) | 4 | |
| % Alignment | 97.08% | |

**Figure S2.B.** The second intron lengths in *Hipposideros armiger* and *H. pendleburyi* are 134 bp and 137 bp, respectively. This difference reduces the alignment percentage to 97.08%. (https://www.genome.jp/tools-bin/clustalw).

Third intron

*Hipposideros armiger*

>armigerintron3 (578 pb)

GTGAGTGGCCCCTTCTGGGTTGGAGGGGCTGATGGGATGTAGTGCATGGAACTTCCTATAGATCAGTTAACCATCTAGGGCAGAGAAGGCTCTTTCTACCCTGGGCTCCTCCCATCACCTGAACCCCACGGGGACTGGCTCCGCAATCTCTTAGAGTAATGGTTCTCAAAGTGTGGTCTTCACCTGGGAACTTGTCAGAAATGCAAATTCTCAGTCCCTAGTCAGAATCTGCATGGAGCCCAGCAGTTTGGATTTAACAAGCCATCCAGAAGATTCTGACTCTTGCTGAAGCTTGAGAGCCCTGACTTAGAGTTTTGAGCTTTCAGCCCATGCTCCAGTCTCAATTTTGCTGTGATGGGCTGTGTGACCCTGGGAGCCCGTAGCCATCTCTGGGCTTCAGTTTCTTCATCTGTTTATGGCTATAGGGATTTACCACATGCTCCAAAGTTCACAGCCAGAGGGTGGACTGGAGCCCCAAGGCTCCTAGGGTGGCCCAGGAAGGGGGGTGTCTAGTTGGGGGGGGGGTCTTGACCCTGGGTCCAGCTCCCACAAGGAACCTGTTTCCTCTCGGTGCACAG

*Hipposideros pendleburyi*

>pendleburyintron3 (574 pb)

GTGAGTGGCCCCTTCTGGGTTGGAGGGGCTGATGGGATGTGGTGCATGGAACTTCCTATAGATCAGTTAACCATCTAGGGCAGAGAAGGCTCCTTCTACCTGGGCTGCTCCCATCACCTGAACCCCACGGGGACTGGCTCCGCAATCTCTTAGAGTAATGGTTCTCAAAGTGTGGTCTTCACCTGGGAACTTGTCAGAAATGCAAATTCTCAGTCCCTAGTCAGAATCTGCATGGAGCCCAGCAATTTGGATTTAACAAGCCATCCAGAAGATTCTGACTCTTGCTGAAGCTTGAGAGCCCTGACTTAGAGTTTTGAGCTTTCAGCCCATGCTCCAGTCTCAATTTTGCTGTGATGGGCTGTGTGACCCTGGGAGCCCGTAGCCATCTCTGGGCTTCAGTTTCTTCATCTGTTTATGGCTATCGGGATTTACCACATGCTCCAAAGTTCACAGCCAGAGGGTGGACTGGAGCCCCAAGGCTCCTAGGGTGGCCCAGGAAGGGGGGTGTCTAGTTGGGGGGGTCTTGACCCTGGGTCCAGCTCCCACAAGGAACCTGTTTCCTCTCGGTGCACAG

Alignment:


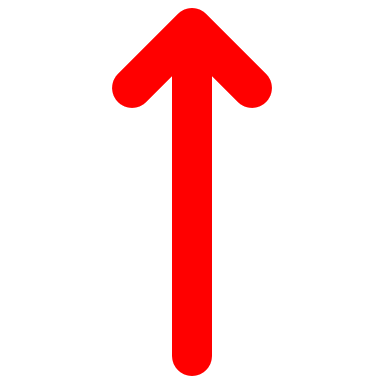

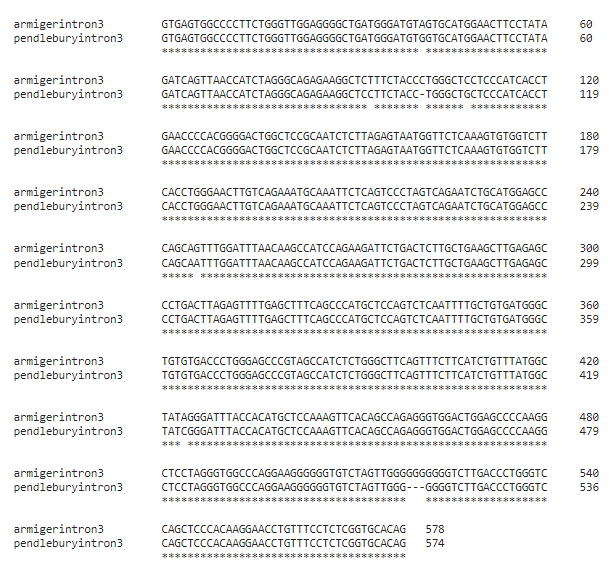

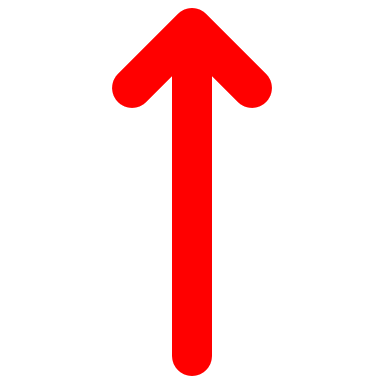

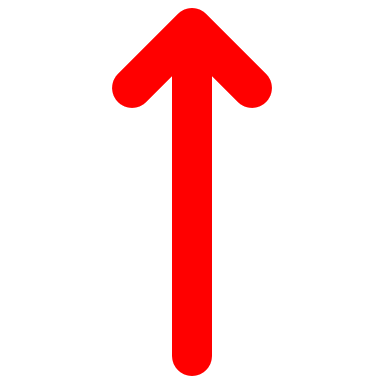

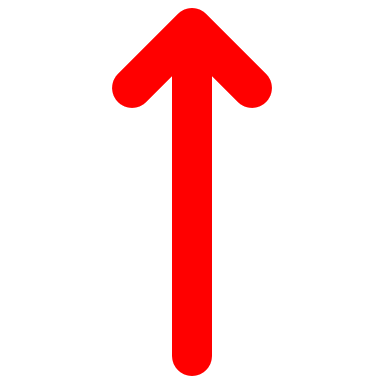

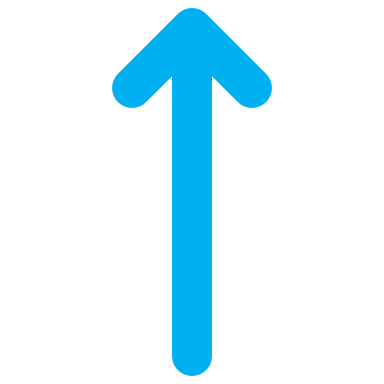

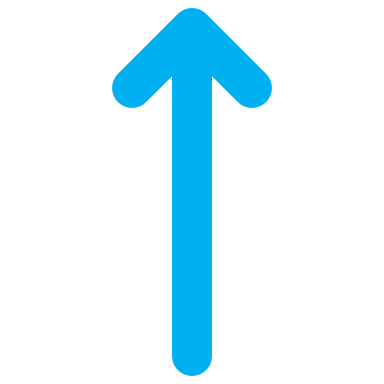

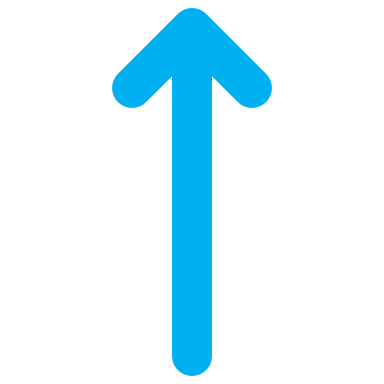


| Intron 3 | *Hipposideros armiger* | *Hipposideros pendleburyi* |
| --- | --- | --- |
| Length (bp) | 578 | 574 |
| Differences (bp) | 4 (Length) + 3 (Sequence) | |
| % Alignment | 98.44% | |

**Figure S2.C.** The third intron in Hipposideros armiger and H. pendleburyi exhibits differences in both length (indicated by 4 red arrows) and sequence (highlighted by 3 blue arrows). Nonetheless, the alignment percentage remains at 98.44%. (https://www.genome.jp/tools-bin/clustalw).
