# Supplementary material for: Genome Mining and Structural Study of Cathelicidins Across Chiroptera Species
Source: Biochem Res Int. 2025 Sep 23;2025:5461549. doi: 10.1155/bri/5461549 (PMC12483743; doi:10.1155/bri/5461549)
Supplement: Supporting Information 3 — Figure S3: This figure shows the Van der Waals interaction analysis of the conserved and modified domains. [file 5461549.f3.docx]

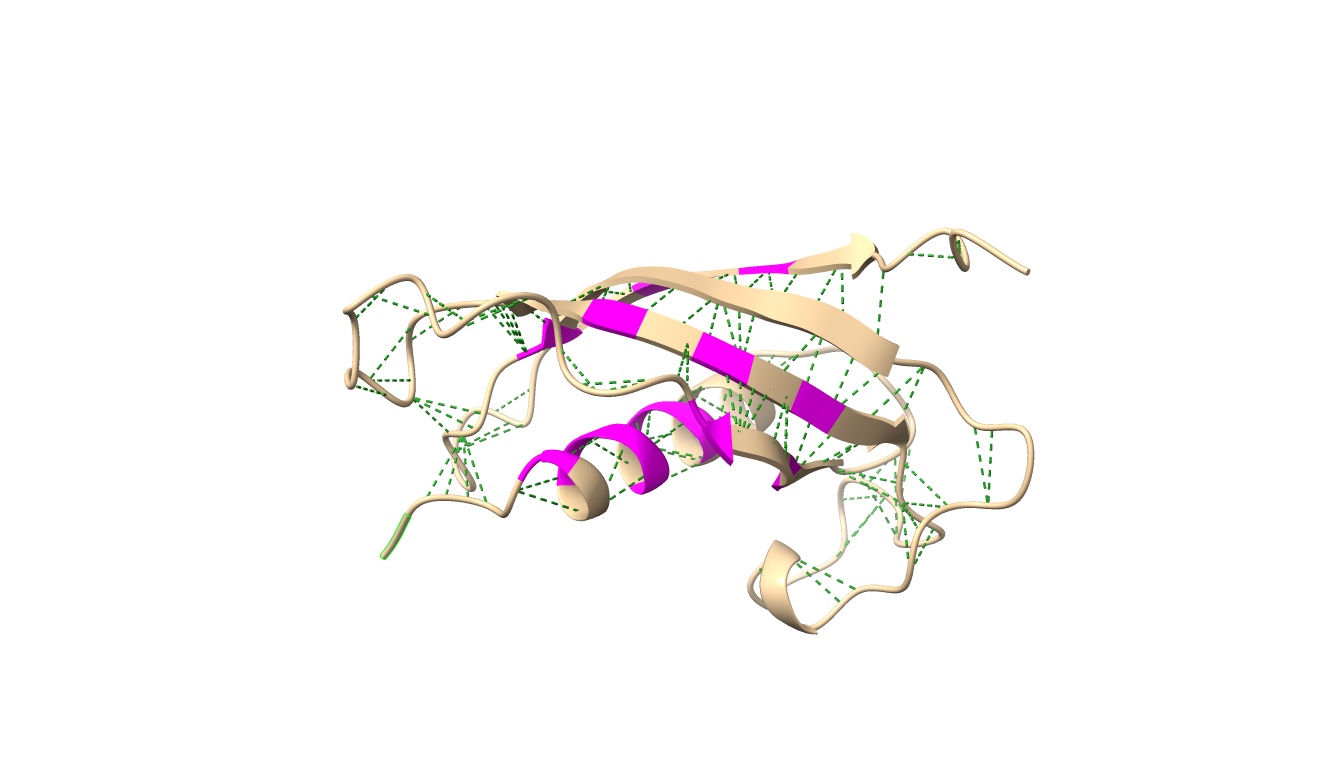

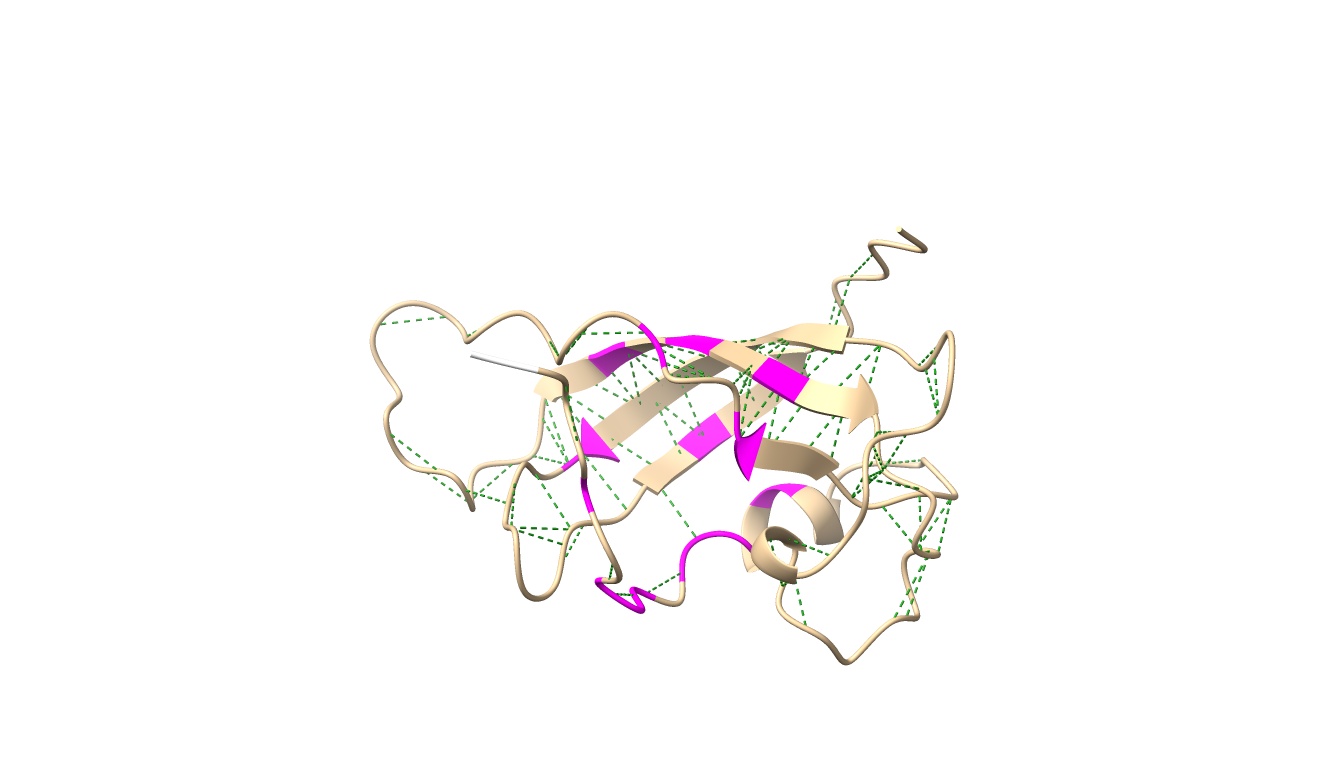


**Q**

**Q**

**V**

**V**

| >Consensus_sequence_Conserved_domain_  QALS**Y**NE**AVL**R**AV**DG**F**NQRSSEASLYR**L**LE**L**DPPQRPDGDDNPNTPKP**V**S**F**T**V**KETVCPRTTQLPPEQCDFKENGLVKQCAGT**V**TLDQANGYFD**I**N**C**AEEIQKV | >Consensus_sequence_Conserved domain_Glicine  QALS**G**NE**ggg**R**gg**DG**g**NQRSSEASLYR**g**LE**g**DPPQRPDGDDNPNTPKP**g**S**g**T**g**KETVCPRTTQLPPEQCDFKENGLVKQCAGT**g**TLDQANGYFD**g**NCAEEIQKV |
| --- | --- |

**Figure S3. Van der Waals Interaction Analysis**. Using the FASTA sequences of the conserved and modified domains, two β-sheet models were generated via the AlphaFold Colab platform and analysed using UCSF ChimeraX. Model (A) shows the conserved domain with Van der Waals interactions, highlighting 15 critical amino acids (purple) identified by Sánchez et al. (39) as essential for β-sheet folding. In model (B), these residues were replaced with glycine, altering the Van der Waals interactions and disrupting proper folding, including partial loss of the α-helix in the structure.
