# Supplementary material for: Genome Mining and Structural Study of Cathelicidins Across Chiroptera Species
Source: Biochem Res Int. 2025 Sep 23;2025:5461549. doi: 10.1155/bri/5461549 (PMC12483743; doi:10.1155/bri/5461549)
Supplement: Supporting Information 4 — Figure S4: Representation of potential recombinant bat cathelicidins. [file 5461549.f4.docx]

**Peptides**

Type 7

Type 4


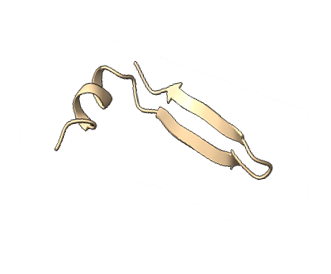

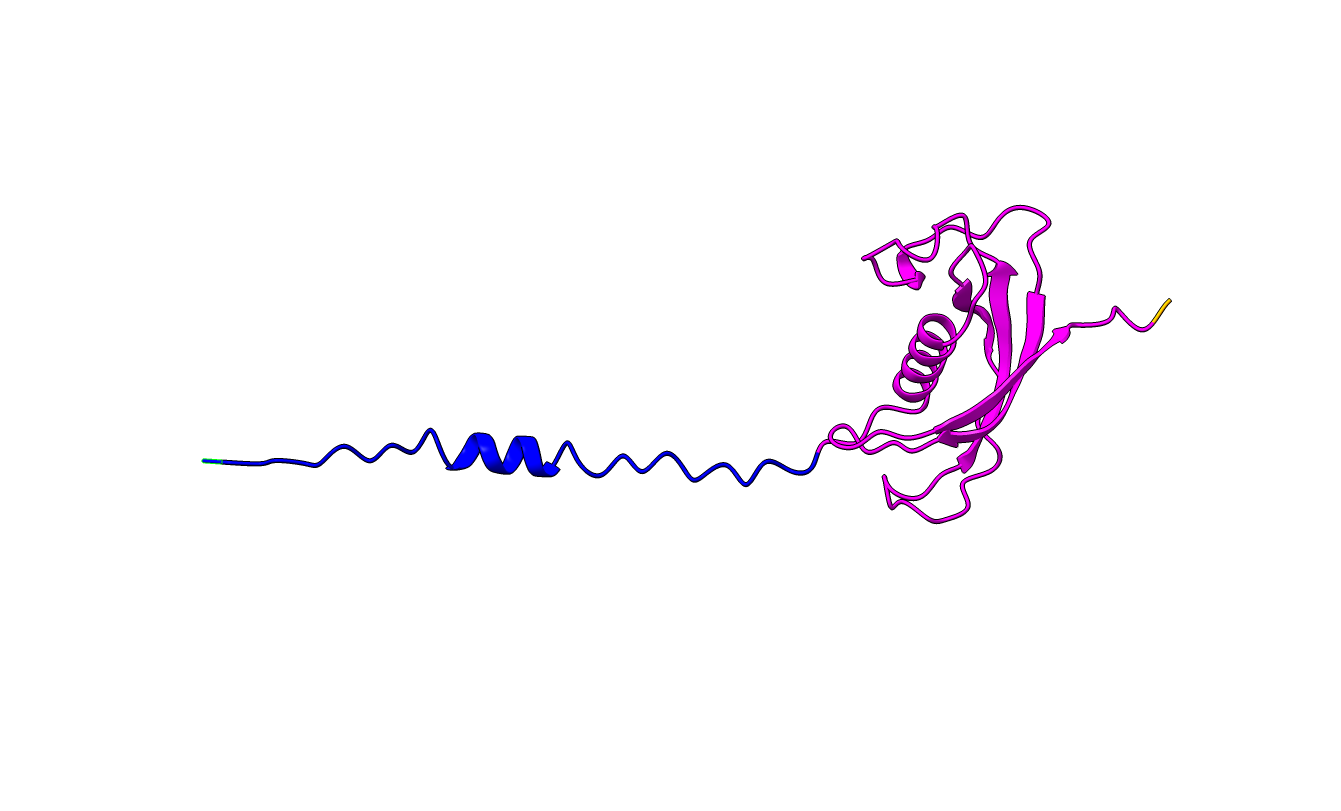

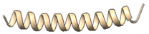

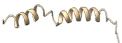

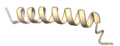

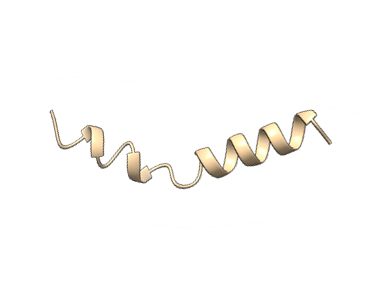

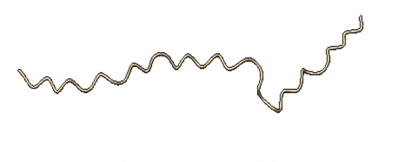

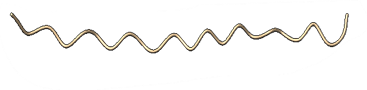

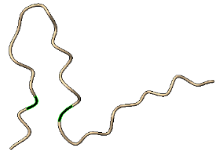


**Recombinant signal peptide**

**Recombinant Conserved Domain**

Type 1

Type 2

Type 3

Type 5

Type 6

Type 8

**V**

**M**

>Recombinant_bat_Cathelicidin_Sequence

METQRDSLPWGRRSWSLLLLLLGLAVPPATAAAAQALSYNEAVLRAVDGFNQRSSEASLYRLLELDPPQRPDGDDNPNTPKPVSFTVKETVCPRTTQLPPEQCDFKENGLVKQCAGTVTLDQANGYFDINCAEEIQKV

**Figure S4.** Representation of potential recombinant bat cathelicidins, showing the consensus FASTA sequences of the signal peptide (blue) and the conserved cathelicidin domain (purple). The recombinant protein was designed by fusing a consensus signal peptide, derived from the alignment of 72 cathelicidin sequences (data not shown), with the consensus sequence of the conserved domain, as identified in the percent identity diagram. This construct can be further fused with any of the eight peptide variants to generate recombinant proteins of interest for future studies.
