# Supplementary material for: Genome Mining and Structural Study of Cathelicidins Across Chiroptera Species
Source: Biochem Res Int. 2025 Sep 23;2025:5461549. doi: 10.1155/bri/5461549 (PMC12483743; doi:10.1155/bri/5461549)
Supplement: Supporting Information 5 — Table S1: Summary of the cathelicidins, incomplete sequences, nonfunctional cathelicidins and antimicrobial peptides (AMPs) identified in the analysis of 62 NCBI Reference Sequences. [file 5461549.f5.docx]

| **Family: Vespertilionidae** | | **Cathelicidin** | **Peptides** | | **NCBI Reference Sequence (DNA)** | | **Accession number (Protein)** |
| --- | --- | --- | --- | --- | --- | --- | --- |
| *Pipistrellus kuhlii* | | 1 | 1 | | NW_023425416.1 | | KAF6334921.1 |
| *Pipistrellus pipistrellus* | | 1 | 1 | | LR862370.1 | | No |
| *Myotis lucifugus* | | 4 | 4 | | NW_005873091.1, NW_005874380.1 | | XP_006108360.2, XP_006108361.1, XP_006108362.1, XP_006108800.3 |
| *Murina aurata feae* | | 1 | 1 | | PVJC01074807.1 | | No |
| *Lasiurus borealis* | | 1 | 1 | | PVJN01095030.1 | | No |
| *Eptesicus fuscus* | | 1 | 1 | | NW_007370654.1 | | XP_008154130.1 |
| *Nycticeius humeralis* | | 6 | 6 | | VMDQ010130254.1, VMDQ010138615.1, (VMDQ010262684.1+VMDQ010175972.1),  (VMDQ010262684.1+VMDQ010175277.1),  VMDQ010139221.1, VMDQ010154871.1 | | No |
| *Miniopterus natalensis* | | 1 | 1 | | VMDQ010154871.1 | | XP_016058295.1 |
| *Miniopterus schreibersii* | | 1 | 1 | | PVJG01001731.1 | | No |
| *Myotis brandtii* | | 1 | 1 | | NW_005359310.1 | | EPQ08412.1 |
| *Myotis myotis* | | 2 + 1 Incomplete | 2 | | NW_023416317.1 | | KAF6310192.1, KAF6310193.1 |
| *Myotis davidii* | | 2 Incompletes**** | 1**** | | NW_006295816.1 | | ELK24989.1**** |
| *Aeorestes cinereus* | | 1 | 1 | | JAAGEH010000117.1 | | No |
| **Family: Craseonycteridae** | | **Cathelicidin** | **Peptides** | | **NCBI reference** | | **Accession number** |
| *Craseonycteris thonglongyai* | | 1 | 1 | | PVKE010000134.1 | | No |
| **Family: *Mormoopidae*** | | **Cathelicidin** | **Peptides** | | **NCBI reference** | | **Accession number** |
| *Pteronotus mesoamericanus* | | 6 + 3 non-functional | 9 | | JAIWKQ010000146.1 | | XP_054450801.1 |
| *Mormoops blainvillei* | | 1 | 1 | | PVJD01080913.1 + PVJD01040264.1 | | No |
| **Family: *Molossidae*** | | **Cathelicidin** | **Peptides** | | **NCBI reference** | | **Accession number** |
| *Molossus molossus* | | 1 | 1 | | NW_023425353.1 | | KAF6420041.1 |
| *Tadarida brasiliensis* | | 1 | 1 | | PVIG010095843.1 | | No |
| **Family: *Phyllostomidae*** | | **Cathelicidin** | **Peptides** | | **NCBI reference** | | **Accession number** |
| *Artibeus jamaicensis* | | 3 | 3 | | JAIVGF010000184.1 | | XP_053525262.1, XP_053525356.1, XP_053525371.1 |
| *Carollia perspicillata* | | 1 | 1 | | PVKM010224682 + PVKM010183853.1 | | No |
| *Desmodus rotundus *** | | 5** | 3** | | CM040281.1 + NC_071394.1,  NC_071394.1, CM040281.1 | | XP_053786266.1, XP_024421797.2, |
| *Micronycteris hirsuta* | | 3 | 3 | | PVJI01021093.1 | | No |
| *Anoura caudifer* | | 1 | 1 | | PVKU01026301 | | No |
| *Phyllostomus discolor* | | 6 | 6 | | NC_040909.2, RXPB02004958.1 | | XP_028374415.1, KAF6098810.1 |
| *Phyllostomus hastatus* | | 5 | 5 | | NW_025334009.1 | | XP_045712867.1, XP_045713206.1 |
| *Tonatia saurophila* | | 2 | 2 | | PVIA01020748.1, PVIA01018809.1 | | No |
| *Sturnira hondurensis* | | 1 | 1 | | NW_023512848.1 | | XP_036905727.1 |
| *Macrotus californicus* | | 1 | 0 | | VMDR010105903.1, VMDR010252616.1, VMDR010000046.1 | | No |
| *Trachops cirrhosus* | | 2 + 4 Incompletes | 6 | | JAPYXV010010172.1, JAPYXV010039841.1  JAPYXV010011263.1, JAPYXV010022013.1,  JAPYXV010000007.1 | | No |
| **Family: *Pteropodidae*** | | **Cathelicidin** | **Peptides** | | **NCBI reference** | | **Accession number** |
| *Rousettus aegyptiacus* | | 1 | 1 | | NW_023416307.1 | | KAF6473429.1 |
| *Eonycteris spelaea* | | 0 + 1 non-functional | 1 | | PUFA01000145.1 | | No |
| *Cynopterus brachyotis* | | 1 | 1 | | SSHV01005765.1 | | No |
| *Macroglossus sobrinus* | | 1 | 1 | | PVKZ01005166.1 | | No |
| *Eidolon dupreanum* | | 1 | 1 | | CM053507.1 | | No |
| *Eidolon helvum* | | 1 | 1 | | KE779879.1 | | No |
| *Pteropus giganteus* | | 1 | 1 | | NW_024349803.1 | | XP_039693580.1 |
| **Family: *Rhinolophidae*** | | **Cathelicidin** | **Peptides** | | **NCBI reference** | | **Accession number** |
| *Rhinolophus ferrumequinum* | | 1 | 1 | | NC_046300.1 | | XP_032988513.1 |
| *Rhinolophus sinicus* | | 1 | 1 | | NW_017738954.1 | | XP_019579268.1 |
| **Family: *Hipposideridae*** | | **Cathelicidin** | **Peptides** | | **NCBI reference** | | **Accession number** |
| *Hipposideros armiger* | | 1* | 1* | | NW_017731368.1 | | XP_019486615.1 |
| *Hipposideros pendleburyi* | | 1* | 1* | | JAHQIX010028625.1 | | XP_019486615.1 |
| *Hipposideros galeritus* | | 1 | 1 | | PVLB01023204.1 | | No |
| **Total families** | **Total Species** | **Total** | **Total** | | **Total (NCBI Reference Sequence)** | | **Total (Accession number)** |
| 8 | 41 | 72 completes + 7 incompletes + 4 non-functional | 78***  53 novel sequences  25 NCBI sequences | | 62 | | 28 Completes + 1**** Incomplete cathelicidins in NCBI Database |
| **Summary** | | | | | | | |
| **Cathelicidins** | | **Completes sequences** | | **Incompletes sequences** | | **Non-functional sequences** | |
| Total sequences: **(n = 83)** | | **72** | | **7** | | **4** | |
| Novel sequences (n = 54) | | 44 | | 6 | | 4 | |
| NCBI sequences (n = 29) | | 28 | | 1 | | 0 | |
| **Peptides** | | **Derived from completes Cathelicidins** | | **Derived from incompletes sequences** | | **Derived from non-functional sequences** | |
| Total sequences: **(n = 78)** | | **69** | | **5** | | **4** | |
| Novel sequences (n = 53) | | 44 | | 5 | | 4 | |
| NCBI sequences (n = 25) | | 25 | | 0 | | 0 | |

**Table S1.** Complete cathelicidin sequences, incomplete sequences, non-functional cathelicidins, and antimicrobial peptides (AMPs) identified from 62 NCBI Reference Sequences. Accession numbers are provided for previously reported sequences, whereas novel sequences are listed without accession numbers.

* Cathelicidins are identical in both nucleotide sequence and peptide product.

** Desmodus rotundus encodes five distinct cathelicidins, comprising two pairs that share identical peptide sequences but differ in their N-terminal protein regions, resulting in a total of five cathelicidins and three unique peptides.

*** Seventy-eight unique peptides were identified: 69 from 72 complete cathelicidins (including one peptide shared by Hipposideros armiger and H. pendleburyi, and three—rather than five—from D. rotundus), five from incomplete cathelicidins (four from Trachops cirrhosus and one from Myotis davidii), and four from non-functional cathelicidins (three from Phyllostomus mesoamericanus and one from Eonycteris spelaea).

**** Two incomplete cathelicidins were retrieved from the M. davidii scaffold, one of which had already been deposited in the NCBI database despite lacking the peptide sequence.

The table additionally summarizes the total number of sequences recovered from the analysis of the 62 NCBI Reference Sequences.
