# Supplementary material for: Genome Mining and Structural Study of Cathelicidins Across Chiroptera Species
Source: Biochem Res Int. 2025 Sep 23;2025:5461549. doi: 10.1155/bri/5461549 (PMC12483743; doi:10.1155/bri/5461549)
Supplement: Supporting Information 6 — Table S2: Summary of NCBI reference sequences of genomic DNA (gDNA) used for mining. It details the positions of cathelicidins' UTRs and exon sequences in base pairs (bp) from the start of the chromosome, contig or scaffold, along with sequence lengths. [file 5461549.f6.docx]

| **Reference Sequences** | **Cathelicidins' genomic location (base pair length)** | | | | | | | | | | | |
| --- | --- | --- | --- | --- | --- | --- | --- | --- | --- | --- | --- | --- |
| NW_023425416.1 | (Reverse) PolA – | | (Reverse) Exon 4- | | (Reverse) Exon 3- | | (Reverse) Exon 2- | | (Reverse) Exon 1 - | | (Reverse) TATA Box - | |
| *Pipistrellus kuhlii* | Start | End | Start | End | Start | End | Start | End | Start | End | Start | End |
|  | 52476834 | 52476829 | 52477148 | 52476876 | 52477600 | 52477529 | 52478338 | 52478231 | 52478825 | 52478613 | 52478878 | 52478874 |
|  | Length | 6 | Length | 273 | Length | 72 | Length | 108 | Length | 213 | Length | 5 |
| LR862370.1 | (Reverse) PolA – | | (Reverse) Exon 4- | | (Reverse) Exon 3- | | (Reverse) Exon 2- | | (Reverse) Exon 1 - | | (Reverse) TATA Box - | |
| *Pipistrellus pipistrellus* | Start | End | Start | End | Start | End | Start | End | Start | End | Start | End |
|  | 51285207 | 51285212 | 51285354 | 51285605 | 51285998 | 51286069 | 51286285 | 51286392 | 51286808 | 51287021 | 51287068 | 51287073 |
|  | Length | 6 | Length | 252 | Length | 72 | Length | 108 | Length | 213 | Length | 5 |
| NW_005873091.1 | (Reverse) PolA – | | (Reverse) Exon 4- | | (Reverse) Exon 3- | | (Reverse) Exon 2- | | (Reverse) Exon 1 - | | (Reverse) TATA Box - | |
| *Myotis lucifugus* | Start | End | Start | End | Start | End | Start | End | Start | End | Start | End |
|  | 4106 | 4101 | 4286 | 4167 | 4860 | 4789 | 5087 | 4980 | 6271 | 6068 | 6316 | 6310 |
|  | Length | 6 | Length | 120 | Length | 72 | Length | 108 | Length | 204 | Length | 7 |
| NW_005873091.1 | (Reverse) PolA – | | (Reverse) Exon 4- | | (Reverse) Exon 3- | | (Reverse) Exon 2- | | (Reverse) Exon 1 - | | (Reverse) TATA Box - | |
| *Myotis lucifugus* | Start | End | Start | End | Start | End | Start | End | Start | End | Start | End |
|  | 13984 | 13979 | 14165 | 14055 | 14733 | 14662 | 14959 | 14852 | 16077 | 15874 | 16122 | 16116 |
|  | Length | 6 | Length | 111 | Length | 72 | Length | 108 | Length | 204 | Length | 7 |
| NW_005873091.1 | (Reverse) PolA – | | (Reverse) Exon 4- | | (Reverse) Exon 3- | | (Reverse) Exon 2- | | (Reverse) Exon 1 - | | (Reverse) TATA Box - | |
| *Myotis lucifugus* | Start | End | Start | End | Start | End | Start | End | Start | End | Start | End |
|  | 23893 | 23888 | 24073 | 23933 | 24646 | 24575 | 24872 | 24765 | 26114 | 25911 | 26157 | 26151 |
|  | Length | 6 | Length | 141 | Length | 72 | Length | 108 | Length | 204 | Length | 7 |
| NW_005874380.1 | (Forward) TATA Box | | (Forward) Exon 1 + | | (Forward) Exon 2 + | | (Forward) Exon 3 + | | (Forward) Exon 4 + | | (Forward) PolA + | |
| *Myotis lucifugus* | Start | End | Start | End | Start | End | Start | End | Start | End | Start | End |
|  | 5324 | 5331 | 5368 | 5371 | 6498 | 6605 | 6725 | 6796 | 7295 | 7417 | 7469 | 7474 |
|  | Length | 7 | Length | 204 | Length | 108 | Length | 72 | Length | 123 | Length | 6 |
| PVJC01074807.1 | (Forward) TATA Box | | (Forward) Exon 1 + | | (Forward) Exon 2 + | | (Forward) Exon 3 + | | (Forward) Exon 4 + | | (Forward) PolA + | |
| *Murina aurata feae* | Start | End | Start | End | Start | End | Start | End | Start | End | Start | End |
|  | 825 | 832 | 871 | 1074 | 1952 | 2059 | 2182 | 2253 | 2759 | 2878 | 3067 | 3072 |
|  | Length | 8 | Length | 204 | Length | 108 | Length | 72 | Length | 120 | Length | 6 |
| PVJN01095030.1 | (Forward) TATA Box | | (Forward) Exon 1 + | | (Forward) Exon 2 + | | (Forward) Exon 3 + | | (Forward) Exon 4 + | | (Forward) PolA + | |
| *Laisiurus borealis* | Start | End | Start | End | Start | End | Start | End | Start | End | Start | End |
|  | 94 | 101 | 141 | 350 | 1528 | 1635 | 1755 | 1826 | 2351 | 2509 | 2559 | 2564 |
|  | Length | 8 | Length | 210 | Length | 108 | Length | 72 | Length | 159 | Length | 6 |
| NW_007370654.1 | (Reverse) PolA – | | (Reverse) Exon 4- | | (Reverse) Exon 3- | | (Reverse) Exon 2- | | (Reverse) Exon 1 - | | (Reverse) TATA Box - | |
| *Eptesicus fuscus* | Start | End | Start | End | Start | End | Start | End | Start | End | Start | End |
|  | 40006638 | 40006633 | 40006910 | 40006767 | 40007527 | 40007456 | 40007752 | 40007645 | 40009000 | 40008797 | 40009046 | 40009039 |
|  | Length | 6 | Length | 144 | Length | 72 | Length | 108 | Length | 204 | Length | 8 |
| VMDQ010154871.1 | (Forward) TATA Box | | (Forward) Exon 1 + | | (Forward) Exon 2 + | | (Forward) Exon 3 + | | (Forward) Exon 4 + | | (Forward) PolA + | |
| *Nycticeius humeralis* | Start | End | Start | End | Start | End | Start | End | Start | End | Start | End |
|  | 454 | 461 | 500 | 691 | 1622 | 1729 | 1817 | 1888 | 2318 | 2506 | 2606 | 2611 |
|  | Length | 8 | Length | 82 | Length | 108 | Length | 72 | Length | 189 | Length | 6 |
| VMDQ010139221.1 | (Reverse) PolA – | | (Reverse) Exon 4- | | (Reverse) Exon 3- | | (Reverse) Exon 2- | | (Reverse) Exon 1 - | | (Reverse) TATA Box - | |
| *Nycticeius humeralis* | Start | End | Start | End | Start | End | Start | End | Start | End | Start | End |
|  | 754 | 750 | 1016 | 855 | 1572 | 1501 | 1776 | 1669 | 2891 | 2703 | 2935 | 2929 |
|  | Length | 6 | Length | 162 | Length | 72 | Length | 108 | Length | 189 | Length | 8 |
| VMDQ010130254.1 | (Reverse) PolA – | | (Reverse) Exon 4- | | (Reverse) Exon 3- | | (Reverse) Exon 2- | | (Reverse) Exon 1 - | | (Reverse) TATA Box - | |
| *Nycticeius humeralis* | Start | End | Start | End | Start | End | Start | End | Start | End | Start | End |
|  | 685 | 680 | 950 | 786 | 1448 | 1377 | 1643 | 1536 | 2761 | 2570 | 2807 | 2800 |
|  | Length | 6 | Length | 165 | Length | 72 | Length | 108 | Length | 192 | Length | 8 |
| VMDQ010175972.1 | (Forward) TATA Box | | (Forward) Exon 1 + | | (Forward) Exon 2 + | | (Forward) Exon 3 + | | (Forward) Exon 4 + | | (Forward) PolA + | |
| *Nycticeius humeralis* | Start | End | Start | End | Start | End | Start | End | Start | End | Start | End |
|  |  |  |  |  | 506 | 613 | 710 | 781 | 1231 | 1398 | 1462 | 1467 |
|  | Length | ? | Length | ? | Length | 108 | Length | 72 | Length | 168 | Length | 6 |
| VMDQ010138615.1 | (Reverse) PolA – | | (Reverse) Exon 4- | | (Reverse) Exon 3- | | (Reverse) Exon 2- | | (Reverse) Exon 1 - | | (Reverse) TATA Box - | |
| *Nycticeius humeralis* | Start | End | Start | End | Start | End | Start | End | Start | End | Start | End |
|  | 1675 | 1670 | 1921 | 1757 | 2480 | 2409 | 2675 | 2568 |  |  |  |  |
|  | Length | 6 | Length | 165 | Length | 72 | Length | 108 | Length | ? | Length | ? |
| VMDQ010175972.1 | (Forward) TATA Box | | (Forward) Exon 1 + | | (Forward) Exon 2 + | | (Forward) Exon 3 + | | (Forward) Exon 4 + | | (Forward) PolA + | |
| *Nycticeius humeralis* | Start | End | Start | End | Start | End | Start | End | Start | End | Start | End |
|  |  |  |  |  | 516 | 623 | 711 | 782 | 1212 | 1400 | 1482 | 1487 |
|  | Length | ? | Length | ? | Length | 108 | Length | 72 | Length | 189 | Length | 6 |
| NW_015504329.1 | (Forward) TATA Box | | (Forward) Exon 1 + | | (Forward) Exon 2 + | | (Forward) Exon 3 + | | (Forward) Exon 4 + | | (Forward) PolA + | |
| *Miniopterus natalensis* | Start | End | Start | End | Start | End | Start | End | Start | End | Start | End |
|  | 1209435 | 1209441 | 1209478 | 1209675 | 1210298 | 1210405 | 1210520 | 1210591 | 1211183 | 1211320 | 1211361 | 1211366 |
|  | Length | 7 | Length | 198 | Length | 108 | Length | 72 | Length | 138 | Length | 6 |
| PVJG01001731.1 | (Forward) TATA Box | | (Forward) Exon 1 + | | (Forward) Exon 2 + | | (Forward) Exon 3 + | | (Forward) Exon 4 + | | (Forward) PolA + | |
| *Miniopterus schreibersii* | Start | End | Start | End | Start | End | Start | End | Start | End | Start | End |
|  | 1209435 | 1209441 | 1209478 | 1209675 | 1210298 | 1210405 | 1210520 | 1210591 | 1211183 | 1211320 | 1211361 | 1211366 |
|  | Length | 7 | Length | 198 | Length | 108 | Length | 72 | Length | 138 | Length | 6 |
| NW_005359310.1 | (Forward) TATA Box | | (Forward) Exon 1 + | | (Forward) Exon 2 + | | (Forward) Exon 3 + | | (Forward) Exon 4 + | | (Forward) PolA + | |
| *Myotis brandtii* | Start | End | Start | End | Start | End | Start | End | Start | End | Start | End |
|  | 883814 | 883820 | 883858 | 884061 | 885036 | 885143 | 885263 | 885334 | 885844 | 885984 | 886022 | 886025 |
|  | Length | 6 | Length | 204 | Length | 108 | Length | 72 | Length | 141 | Length | 4 |
| NW_006295816.1 | (Forward) TATA Box | | (Forward) Exon 1 + | | (Forward) Exon 2 + | | (Forward) Exon 3 + | | (Forward) Exon 4 + | | (Forward) PolA + | |
| *Myotis davidii* | Start | End | Start | End | Start | End | Start | End | Start | End | Start | End |
|  | 344818 |  | 344862 | 345065 | 346055 | 346162 | 366282 | 346371 |  |  | 349027 |  |
|  | Length | ? | Length | 204 | Length | 108 | Length | 90 | Length | ? | Length | ? |
| NW_006295816.1 | (Forward) TATA Box | | (Forward) Exon 1 + | | (Forward) Exon 2 + | | (Forward) Exon 3 + | | (Forward) Exon 4 + | | (Forward) PolA + | |
| *Myotis davidii* | Start | End | Start | End | Start | End | Start | End | Start | End | Start | End |
|  |  |  |  |  |  |  | 169 | 240 | 887 | 1027 | 1067 |  |
|  | Length | ? | Length | ? | Length | ? | Length | 72 | Length | 141 | Length | ? |
| NW_023416317.1 | (Reverse) PolA – | | (Reverse) Exon 4- | | (Reverse) Exon 3- | | (Reverse) Exon 2- | | (Reverse) Exon 1 - | | (Reverse) TATA Box - | |
| *Myotis myotis* | Start | End | Start | End | Start | End | Start | End | Start | End | Start | End |
|  | 57665352 | 57665366 | 57665535 | 57665392 | 57666109 | 57666038 | 57666336 | 57666229 | 57667436 | 57667233 | 57667480 | 57667474 |
|  | Length | 6 | Length | 144 | Length | 72 | Length | 108 | Length | 204 | Length | 7 |
| NW_023416317.1 | (Reverse) PolA – | | (Reverse) Exon 4- | | (Reverse) Exon 3- | | (Reverse) Exon 2- | | (Reverse) Exon 1 - | | (Reverse) TATA Box - | |
| *Myotis myotis* | Start | End | Start | End | Start | End | Start | End | Start | End | Start | End |
|  | 57677840 | 57677835 | 57678059 | 57677967 |  |  |  |  | 57719380 | 57719177 | 57719397 | 57719390 |
|  | Length | 6 | Length | 93 | Length | ? | Length | ? | Length | 204 | Length | 7 |
| NW_023416317.1 | (Reverse) PolA – | | (Reverse) Exon 4- | | (Reverse) Exon 3- | | (Reverse) Exon 2- | | (Reverse) Exon 1 - | | (Reverse) TATA Box - | |
| *Myotis myotis* | Start | End | Start | End | Start | End | Start | End | Start | End | Start | End |
|  | 57725639 | 57725634 | 57725822 | 57725679 | 57726395 | 57726324 | 57726621 | 57726514 | 57727903 | 57727700 | 57727947 | 57727941 |
|  | Length | 6 | Length | 144 | Length | 72 | Length | 108 | Length | 204 | Length | 7 |

**Table S2.** Summary of the NCBI reference sequences of the genomic DNA (gDNA) utilized for the mining process. The precise positions of the cathelicidins' untranslated regions (UTRs) and exon DNA sequences, oriented in the forward or reverse direction, are delineated using distances measured in base pairs (bp) from the beginning of the chromosome, contig, or scaffold employed. Additionally, the length of each sequence is indicated in base pairs (bp). The symbol (?) signifies an incomplete gDNA sequence.

| **Reference Sequences** | | **Cathelicidins' genomic location (base pair length)** | | | | | | | | | | | | |
| --- | --- | --- | --- | --- | --- | --- | --- | --- | --- | --- | --- | --- | --- | --- |
| JAAGEH010000117.1 | | (Forward) TATA Box | | | (Forward) Exon 1 + | | (Forward) Exon 2 + | | (Forward) Exon 3 + | | (Forward) Exon 4 + | | (Forward) PolA + | |
| *Aeorestes cinereus* | | Start | | End | Start | End | Start | End | Start | End | Start | End | Start | End |
|  |  | 301852 | | 301859 | 301900 | 302109 | 303386 | 303493 | 303621 | 303692 | 304210 | 304350 | 304389 | 304394 |
|  |  | Length | | 7 | Length | 210 | Length | 108 | Length | 72 | Length | 141 | Length | 6 |
| PVKE010000134.1 | | (Forward) TATA Box | | | (Forward) Exon 1 + | | (Forward) Exon 2 + | | (Forward) Exon 3 + | | (Forward) Exon 4 + | | (Forward) PolA + | |
| *C. thonglongyai* | | Start | | End | Start | End | Start | End | Start | End | Start | End | Start | End |
|  |  | 60146 | | 60153 | 60193 | 60390 | 61027 | 61134 | 61269 | 61340 | 61734 | 61892 | 61930 | 61935 |
|  |  | Length | | 7 | Length | 210 | Length | 108 | Length | 72 | Length | 159 | Length | 6 |
| NW_026555320.1 | | (Forward) TATA Box | | | (Forward) Exon 1 + | | (Forward) Exon 2 + | | (Forward) Exon 3 + | | (Forward) Exon 4 + | | (Forward) PolA + | |
| *Pteronotus mesoamericanus* | | Start | | End | Start | End | Start | End | Start | End | Start | End | Start | End |
|  |  | 3602564 | | 3602568 | 3602610 | 3602811 | 3603414 | 3603522 | 3603632 | 3603734 | 3604336 | 3604458 | 3604500 | 3604505 |
|  |  | Length | | 5 | Length | 201 | Length | 108 | Length | 72 | Length | 123 | Length | 6 |
| NW_026555320.1 | | (Forward) TATA Box | | | (Forward) Exon 1 + | | (Forward) Exon 2 + | | (Forward) Exon 3 + | | (Forward) Exon 4 + | | (Forward) PolA + | |
| *Pteronotus mesoamericanus* | | Start | | End | Start | End | Start | End | Start | End | Start | End | Start | End |
|  |  | 3602564 | | 3602568 | 3602610 | 3602811 | 3603414 | 3603522 | 3603632 | 3603734 | 3604336 | 3604458 | 3604500 | 3604505 |
|  |  | Length | | 5 | Length | 201 | Length | 108 | Length | 72 | Length | 123 | Length | 6 |
| JAIWKQ010000146.1 | **Non-functional** | | (Forward) TATA Box | | (Forward) Exon 1 + | | (Forward) Exon 3 + | | (Forward) Exon 4 + | | (Forward) Exon 4 + | | (Forward) PolA + | |
| *Pteronotus mesoamericanus* |  |  | Start | End | Start | End | Start | End | Start | End | Start | End | Start | End |
|  |  |  | 3611896 |  | 3611943 | 3612143 | 3612997 | 3613068 | 3613196 | 3613306 | 3613680 | 3613814 | 3613921 | 3613926 |
|  |  |  | Length | ? | Length | 201 | Length | 72 | Length | 111 | Length | 135 | Length | 6 |
|  |  |  | (Forward) TATA Box | | (Forward) Exon 1 + | | (Forward) Exon 2 + | | (Forward) Exon 3 + | | (Forward) Exon 4 + | | (Forward) PolA + | |
|  |  |  | Start | End | Start | End | Start | End | Start | End | Start | End | Start | End |
|  |  |  |  |  | 3616075 | 3616182 | 3616075 | 3616182 | 3616325 | 3616396 | 3617003 | 3617059 | 3617147 | 3617153 |
|  |  |  | Length |  | Length | 171 | Length | 108 | Length | 72 | Length | 57 | Length | 6 |
| NW_026555320.1 | | (Forward) TATA Box | | | (Forward) Exon 1 + | | (Forward) Exon 2 + | | (Forward) Exon 3 + | | (Forward) Exon 4 + | | (Forward) PolA + | |
| *Pteronotus mesoamericanus* | | Start | | End | Start | End | Start | End | Start | End | Start | End | Start | End |
|  |  | 3625566 | |  | 3625613 | 3625813 | 3626417 | 3626524 | 3626665 | 3626736 | 3626863 | 3626973 | 3627489 |  |
|  |  | Length | | ? | Length | 201 | Length | 108 | Length | 72 | Length | 111 | Length | ? |
| NW_026555320.1 | | (Forward) TATA Box | | | (Forward) Exon 1 + | | (Forward) Exon 2 + | | (Forward) Exon 3 + | | (Forward) Exon 4 + | | (Forward) PolA + | |
| *Pteronotus mesoamericanus* | | Start | | End | Start | End | Start | End | Start | End | Start | End | Start | End |
|  |  | 3633951 | |  | 3633997 | 3634197 | 3634801 | 3634908 | 3635050 | 3635121 | 3635248 | 3635358 | 3635876 |  |
|  |  | Length | | ? | Length | 201 | Length | 108 | Length | 72 | Length | 111 | Length | ? |
| NW_026555320.1 | | (Forward) TATA Box | | | (Forward) Exon 1 + | | (Forward) Exon 2 + | | (Forward) Exon 3 + | | (Forward) Exon 4 + | | (Forward) PolA + | |
| *Pteronotus mesoamericanus* | | Start | | End | Start | End | Start | End | Start | End | Start | End | Start | End |
|  |  | 3643127 | | 3643174 | 3643174 | 3643375 | 3643981 | 3644089 | 3644230 | 3644302 | 3644899 | 3645013 | 3645078 | 3645084 |
|  |  | Length | | 47 | Length | 201 | Length | 108 | Length | 72 | Length | 114 | Length | 6 |
| NW_026555320.1 | | (Forward) TATA Box | | | (Forward) Exon 1 + | | (Forward) Exon 2 + | | (Forward) Exon 3 + | | (Forward) Exon 4 + | | (Forward) PolA + | |
| *Pteronotus mesoamericanus* | | Start | | End | Start | End | Start | End | Start | End | Start | End | Start | End |
|  |  | 3652277 | |  | 3652324 | 3652524 | 3653130 | 3653237 | 3653377 | 3653448 | 3654031 | 3654144 | 3654234 | 3654240 |
|  |  | Length | | ? | Length | 201 | Length | 108 | Length | 72 | Length | 114 | Length | 6 |
| NW_026555320.1 | | (Forward) TATA Box | | | (Forward) Exon 1 + | | (Forward) Exon 2 + | | (Forward) Exon 3 + | | (Forward) Exon 4 + | | (Forward) PolA + | |
| *Pteronotus mesoamericanus* | | Start | | End | Start | End | Start | End | Start | End | Start | End | Start | End |
|  |  | 3661676 | | 3661723 | 3661723 | 3661923 | 3662527 | 3662634 | 3662775 | 3662846 | 3663457 | 3663521 | 3663604 | 3666310 |
|  |  | Length | | 47 | Length | 201 | Length | 108 | Length | 72 | Length | 64 | Length | 6 |
| PVJD01080913.1 | | (Forward) TATA Box | | | (Forward) Exon 1 + | | (Forward) Exon 2 + | | (Forward) Exon 3 + | | (Forward) Exon 4 + | | (Forward) PolA + | |
| PVJD01040264.1* | | Start | | End | Start | End | Start | End | Start | End | Start | End | Start | End |
| Scaffolds fusion | | 219 | | 225 | 265 | 1815* | 1202* | 1095* | 995* | 884* | 277* | 155* | 114* | 109* |
| *Mormoops blainvillei* | | Length | | 7 | Length | 201 | Length | 108 | Length | 72 | Length | 123 | Length | 6 |
| NW_023425353.1 | | (Forward) TATA Box | | | (Forward) Exon 1 + | | (Forward) Exon 2 + | | (Forward) Exon 3 + | | (Forward) Exon 4 + | | (Forward) PolA + | |
| *Molossus molossus* | | Start | | End | Start | End | Start | End | Start | End | Start | End | Start | End |
|  |  | 63170595 | | 63170601 | 63170642 | 63170839 | 63171429 | 63171536 | 63171670 | 63171741 | 63172255 | 63172390 | 63172422 | 63172427 |
|  |  | Length | | 7 | Length | 198 | Length | 108 | Length | 72 | Length | 135 | Length | 6 |
| PVIG010095843.1 | | (Forward) TATA Box | | | (Forward) Exon 1 + | | (Forward) Exon 2 + | | (Forward) Exon 3 + | | (Forward) Exon 4 + | | (Forward) PolA + | |
| *Tadarida brasiliensis* | | Start | | End | Start | End | Start | End | Start | End | Start | End | Start | End |
|  |  | 745 | | 740 | 936 | 787 | 1534 | 1463 | 1775 | 1668 | 2582 | 2391 | 2629 | 2622 |
|  |  | Length | | 6 | Length | 150 | Length | 108 | Length | 72 | Length | 141 | Length | 7 |
| JAIVGF010000184.1 | | (Forward) TATA Box | | | (Forward) Exon 1 + | | (Forward) Exon 2 + | | (Forward) Exon 3 + | | (Forward) Exon 4 + | | (Forward) PolA + | |
| *Artibeus jamaicensis* | | Start | | End | Start | End | Start | End | Start | End | Start | End | Start | End |
|  |  | 53935111 | | 53935117 | 53935157 | 53935357 | 53935986 | 53936093 | 53936238 | 53936309 | 53936906 | 53937019 | 53937058 | 53937063 |
|  |  | Length | | 7 | Length | 201 | Length | 108 | Length | 72 | Length | 114 | Length | 7 |
| JAIVGF010000184.1 | | (Forward) TATA Box | | | (Forward) Exon 1 + | | (Forward) Exon 2 + | | (Forward) Exon 3 + | | (Forward) Exon 4 + | | (Forward) PolA + | |
| *Artibeus jamaicensis* | | Start | | End | Start | End | Start | End | Start | End | Start | End | Start | End |
|  |  | 53943181 | | 53943187 | 53943227 | 53943427 | 53944271 | 53944378 | 53944522 | 53944593 | 53945195 | 53945290 | 53945331 | 53945336 |
|  |  | Length | | 7 | Length | 201 | Length | 108 | Length | 72 | Length | 114 | Length | 6 |
| JAIVGF010000184.1 | | (Forward) TATA Box | | | (Forward) Exon 1 + | | (Forward) Exon 2 + | | (Forward) Exon 3 + | | (Forward) Exon 4 + | | (Forward) PolA + | |
| *Artibeus jamaicensis* | | Start | | End | Start | End | Start | End | Start | End | Start | End | Start | End |
|  |  | 53952098 | | 53952104 | 53952144 | 53952344 | 53953189 | 53953296 | 53953440 | 53953511 | 53954115 | 53954216 | 53954257 | 53954262 |
|  |  | Length | | 7 | Length | 201 | Length | 108 | Length | 72 | Length | 102 | Length | 6 |
| PVKM010224682 | | (Reverse) PolA – | | | (Reverse) Exon 4- | | (Reverse) Exon 3- | | (Reverse) Exon 2- | | (Reverse) Exon 1 - | | (Reverse) TATA Box - | |
| PVKM010183853.1 | | Start | | End | Start | End | Start | End | Start | End | Start | End | Start | End |
| Scaffolds fusion | | 1083 | | 1077 | 1037 | 837 | 1824 | 1717 | 1576 | 1505 | 911 | 810 | 767 | 763 |
| *Carollia perspicillata* | | Length | | 7 | Length | 201 | Length | 108 | Length | 72 | Length | 102 | Length | 5 |
| CM040281.1 | | (Forward) TATA Box | | | (Forward) Exon 1 + | | (Forward) Exon 2 + | | (Forward) Exon 3 + | | (Forward) Exon 4 + | | (Forward) PolA + | |
| **Chromosome 8** | | Start | | End | Start | End | Start | End | Start | End | Start | End | Start | End |
| *Desmodus rotundus* | | 129853997 | | 129854003 | 129854043 | 129854243 | 129854773 | 129854880 | 129855033 | 129855104 | 129855681 | 129855794 | 129855833 | 129855838 |
|  |  | Length | | 7 | Length | 201 | Length | 108 | Length | 72 | Length | 114 | Length | 6 |
| CM040281.1 | | (Forward) TATA Box | | | (Forward) Exon 1 + | | (Forward) Exon 2 + | | (Forward) Exon 3 + | | (Forward) Exon 4 + | | (Forward) PolA + | |
| **Chromosome 8** | | Start | | End | Start | End | Start | End | Start | End | Start | End | Start | End |
| *Desmodus rotundus* | | 129864568 | | 129864574 | 129864614 | 129864811 | 129865437 | 129865544 | 129865697 | 129865768 | 129866409 | 129866516 | 129866555 | 129866560 |
|  |  | Length | | 7 | Length | 201 | Length | 108 | Length | 72 | Length | 108 | Length | 6 |
| CM040281.1 | | (Forward) TATA Box | | | (Forward) Exon 1 + | | (Forward) Exon 2 + | | (Forward) Exon 3 + | | (Forward) Exon 4 + | | (Forward) PolA + | |
| **Chromosome 8** | | Start | | End | Start | End | Start | End | Start | End | Start | End | Start | End |
| *Desmodus rotundus* | | 129875298 | | 129875304 | 129875344 | 129875541 | 129876166 | 129876273 | 129876426 | 129876497 | 129877137 | 129877244 | 129877283 | 129877288 |
|  |  | Length | | 7 | Length | 201 | Length | 108 | Length | 72 | Length | 108 | Length | 6 |
| NC_071394.1 | | (Forward) TATA Box | | | (Forward) Exon 1 + | | (Forward) Exon 2 + | | (Forward) Exon 3 + | | (Forward) Exon 4 + | | (Forward) PolA + | |
| *Desmodus rotundus* | | Start | | End | Start | End | Start | End | Start | End | Start | End | Start | End |
|  |  | 129937828 | |  | 129937874 | 129938074 | 129938605 | 129938712 | 129938865 | 129938936 | 129939513 | 129939626 | 129939665 | 53945336 |
|  |  | Length | | ? | Length | 201 | Length | 108 | Length | 72 | Length | 114 | Length | ? |
| NC_071394.1 | | (Forward) TATA Box | | | (Forward) Exon 1 + | | (Forward) Exon 2 + | | (Forward) Exon 3 + | | (Forward) Exon 4 + | | (Forward) PolA + | |
| *Desmodus rotundus* | | Start | | End | Start | End | Start | End | Start | End | Start | End | Start | End |
|  |  | 129948408 | |  | 129948454 | 129948654 | 129949185 | 129949292 | 129949445 | 129949516 | 129950158 | 129950265 | 129950304 |  |
|  |  | Length | | ? | Length | 201 | Length | 108 | Length | 72 | Length | 108 | Length | ? |
| NC_071394.1 | | (Forward) TATA Box | | | (Forward) Exon 1 + | | (Forward) Exon 2 + | | (Forward) Exon 3 + | | (Forward) Exon 4 + | | (Forward) PolA + | |
| *Desmodus rotundus* | | Start | | End | Start | End | Start | End | Start | End | Start | End | Start | End |
|  |  | 129959030 | |  | 129959076 | 129959273 | 129959899 | 129960006 | 129960159 | 129960230 | 129960869 | 129960976 | 129961015 |  |
|  |  | Length | | ? | Length | 201 | Length | 108 | Length | 72 | Length | 108 | Length | ? |

**Table S2.** Summary of the NCBI reference sequences of the genomic DNA (gDNA) utilized for the mining process. The precise positions of the cathelicidins' untranslated regions (UTRs) and exon DNA sequences, oriented in the forward or reverse direction, are delineated using distances measured in base pairs (bp) from the beginning of the chromosome, contig, or scaffold employed. Additionally, the length of each sequence is indicated in base pairs (bp). The symbol (?) signifies an incomplete gDNA sequence.

| **Reference Sequences** | **Cathelicidins' genomic location (base pair length)** | | | | | | | | | | | |
| --- | --- | --- | --- | --- | --- | --- | --- | --- | --- | --- | --- | --- |
| PVJI01021093.1 | (Reverse) PolA – | | (Reverse) Exon 4- | | (Reverse) Exon 3- | | (Reverse) Exon 2- | | (Reverse) Exon 1 - | | (Reverse) TATA Box - | |
| *Micronycteris hirsuta* | Start | End | Start | End | Start | End | Start | End | Start | End | Start | End |
|  | 142 | 138 | 279 | 214 | 944 | 873 | 1192 | 1085 | 1921 | 1721 | 1967 | 1961 |
|  | Length | 6 | Length | 66 | Length | 72 | Length | 108 | Length | 201 | Length | 7 |
| PVJI01021093.1 | (Reverse) PolA – | | (Reverse) Exon 4- | | (Reverse) Exon 3- | | (Reverse) Exon 2- | | (Reverse) Exon 1 - | | (Reverse) TATA Box - | |
| *Micronycteris hirsuta* | Start | End | Start | End | Start | End | Start | End | Start | End | Start | End |
|  | 11757 | 11752 | 11925 | 11839 | 12590 | 12519 | 12838 | 12731 | 13581 | 13381 | 2027 | 2021 |
|  | Length | 6 | Length | 87 | Length | 72 | Length | 108 | Length | 201 | Length | 7 |
| PVJI01021093.1 | (Reverse) PolA – | | (Reverse) Exon 4- | | (Reverse) Exon 3- | | (Reverse) Exon 2- | | (Reverse) Exon 1 - | | (Reverse) TATA Box - | |
| *Micronycteris hirsuta* | Start | End | Start | End | Start | End | Start | End | Start | End | Start | End |
|  | 22218 | 22060 | 22217 | 22104 | 22879 | 22808 | 23127 | 23020 | 23856 | 23656 | 23902 | 23896 |
|  | Length | 6 | Length | 114 | Length | 72 | Length | 108 | Length | 201 | Length | 7 |
| PVJI01021093.1 | (Reverse) PolA – | | (Reverse) Exon 4- | | (Reverse) Exon 3- | | (Reverse) Exon 2- | | (Reverse) Exon 1 - | | (Reverse) TATA Box - | |
| *Micronycteris hirsuta* | Start | End | Start | End | Start | End | Start | End | Start | End | Start | End |
|  | 662 | 657 | 797 | 702 | 1459 | 1388 | 1695 | 1588 | 2738 | 2538 | 2784 | 2778 |
|  | Length | 6 | Length | 96 | Length | 72 | Length | 108 | Length | 201 | Length | 7 |
| NC_040909.2 | (Reverse) PolA – | | (Reverse) Exon 4- | | (Reverse) Exon 3- | | (Reverse) Exon 2- | | (Reverse) Exon 1 - | | (Reverse) TATA Box - | |
| **Chromosome 7** | Start | End | Start | End | Start | End | Start | End | Start | End | Start | End |
| *Phyllostomus discolor* | 5451273 | 5451269 | 5451439 | 5451314 | 5451906 | 5451834 | 5452154 | 5452047 | 5453165 | 5452965 | 5453211 | 5453205 |
|  | Length | 6 | Length | 96 | Length | 72 | Length | 108 | Length | 201 | Length | 7 |
| NC_040909.2 | (Reverse) PolA – | | (Reverse) Exon 4- | | (Reverse) Exon 3- | | (Reverse) Exon 2- | | (Reverse) Exon 1 - | | (Reverse) TATA Box - | |
| **Chromosome 7** | Start | End | Start | End | Start | End | Start | End | Start | End | Start | End |
| *Phyllostomus discolor* | 5463533 | 5463528 | 5463739 | 5463593 | 5464407 | 5464336 | 5464655 | 5465548 | 5465666 | 5465466 | 5465712 | 5465706 |
|  | Length | 6 | Length | 147 | Length | 72 | Length | 108 | Length | 201 | Length | 7 |
| RXPB02004958.1 | (Forward) TATA Box | | (Forward) Exon 1 + | | (Forward) Exon 2 + | | (Forward) Exon 3 + | | (Forward) Exon 4 + | | (Forward) PolA + | |
| **Chromosome 7** | Start | End | Start | End | Start | End | Start | End | Start | End | Start | End |
| *Phyllostomus discolor* | 5463533 | 5463528 | 5463739 | 5463593 | 5464407 | 5464336 | 5464655 | 5465548 | 5465666 | 5465466 | 5465712 | 5465706 |
|  | Length | 6 | Length | 147 | Length | 72 | Length | 108 | Length | 201 | Length | 7 |
| RXPB02004958.1 | (Forward) TATA Box | | (Forward) Exon 1 + | | (Forward) Exon 2 + | | (Forward) Exon 3 + | | (Forward) Exon 4 + | | (Forward) PolA + | |
| *Phyllostomus discolor* | Start | End | Start | End | Start | End | Start | End | Start | End | Start | End |
|  | 908833 | 908839 | 908879 | 909079 | 909892 | 909999 | 910140 | 910211 | 910818 | 910955 | 911024 | 911029 |
|  | Length | 7 | Length | 201 | Length | 108 | Length | 72 | Length | 138 | Length | 6 |
| RXPB02004958.1 | (Forward) TATA Box | | (Forward) Exon 1 + | | (Forward) Exon 2 + | | (Forward) Exon 3 + | | (Forward) Exon 4 + | | (Forward) PolA + | |
| *Phyllostomus discolor* | Start | End | Start | End | Start | End | Start | End | Start | End | Start | End |
|  | 921325 | 921331 | 921371 | 921571 | 922195 | 922302 | 922443 | 922514 | 923104 | 923217 | 923256 | 923261 |
|  | Length | 7 | Length | 201 | Length | 108 | Length | 72 | Length | 114 | Length | 6 |
| RXPB02004958.1 | (Forward) TATA Box | | (Forward) Exon 1 + | | (Forward) Exon 2 + | | (Forward) Exon 3 + | | (Forward) Exon 4 + | | (Forward) PolA + | |
| *Phyllostomus discolor* | Start | End | Start | End | Start | End | Start | End | Start | End | Start | End |
|  | 934408 | 934414 | 934454 | 934654 | 935466 | 935573 | 935713 | 935784 | 936373 | 936492 | 936532 | 936537 |
|  | Length | 7 | Length | 201 | Length | 108 | Length | 72 | Length | 120 | Length | 6 |
| RXPB02004958.1 | (Forward) TATA Box | | (Forward) Exon 1 + | | (Forward) Exon 2 + | | (Forward) Exon 3 + | | (Forward) Exon 4 + | | (Forward) PolA + | |
| *Phyllostomus discolor* | Start | End | Start | End | Start | End | Start | End | Start | End | Start | End |
|  | 946866 | 946872 | 946912 | 947112 | 947925 | 948032 | 948173 | 948244 | 948841 | 948933 | 948973 | 984978 |
|  | Length | 7 | Length | 201 | Length | 108 | Length | 72 | Length | 93 | Length | 6 |
| RXPB02004958.1 | (Forward) TATA Box | | (Forward) Exon 1 + | | (Forward) Exon 2 + | | (Forward) Exon 3 + | | (Forward) Exon 4 + | | (Forward) PolA + | |
| *Phyllostomus discolor* | Start | End | Start | End | Start | End | Start | End | Start | End | Start | End |
|  | 957796 | 957802 | 957842 | 958042 | 958853 | 958960 | 959101 | 959172 | 959569 | 959694 | 959734 | 959739 |
|  | Length | 7 | Length | 201 | Length | 108 | Length | 72 | Length | 126 | Length | 6 |
| NW_025334009.1 | (Reverse) PolA – | | (Reverse) Exon 4- | | (Reverse) Exon 3- | | (Reverse) Exon 2- | | (Reverse) Exon 1 - | | (Reverse) TATA Box - | |
| *Phyllostomus hastatus* | Start | End | Start | End | Start | End | Start | End | Start | End | Start | End |
|  | 5342221 | 5342216 | 5342353 | 5342277 | 5343019 | 5342948 | 5343266 | 5343159 | 5344274 | 5344074 | 5344321 | 5344315 |
|  | Length | 6 | Length | 77 | Length | 72 | Length | 108 | Length | 201 | Length | 7 |
| NW_025334009.1 | (Reverse) PolA – | | (Reverse) Exon 4- | | (Reverse) Exon 3- | | (Reverse) Exon 2- | | (Reverse) Exon 1 - | | (Reverse) TATA Box - | |
| *Phyllostomus hastatus* | Start | End | Start | End | Start | End | Start | End | Start | End | Start | End |
|  | 5354523 | 5354518 | 5354688 | 5354563 | 5355157 | 5355086 | 5355405 | 5355298 | 5356419 | 5356219 | 5356465 | 5356459 |
|  | Length | 6 | Length | 126 | Length | 72 | Length | 108 | Length | 201 | Length | 7 |
| NW_025334009.1 | (Reverse) PolA – | | (Reverse) Exon 4- | | (Reverse) Exon 3- | | (Reverse) Exon 2- | | (Reverse) Exon 1 - | | (Reverse) TATA Box - | |
| *Phyllostomus hastatus* | Start | End | Start | End | Start | End | Start | End | Start | End | Start | End |
|  | 5366765 | 5366760 | 5366908 | 5366825 | 5367378 | 5367307 | 5367624 | 5367517 | 5368642 | 5368442 | 5368688 | 5368682 |
|  | Length | 6 | Length | 84 | Length | 72 | Length | 108 | Length | 201 | Length | 7 |
| NW_025334009.1 | (Reverse) PolA – | | (Reverse) Exon 4- | | (Reverse) Exon 3- | | (Reverse) Exon 2- | | (Reverse) Exon 1 - | | (Reverse) TATA Box - | |
| *Phyllostomus hastatus* | Start | End | Start | End | Start | End | Start | End | Start | End | Start | End |
|  | 5380036 | 5380031 | 5380187 | 5380074 | 5380851 | 5380780 | 5381110 | 5381003 | 5381933 | 5381733 | 5381979 | 5381973 |
|  | Length | 6 | Length | 84 | Length | 72 | Length | 108 | Length | 201 | Length | 7 |
| NW_025334009.1 | (Reverse) PolA – | | (Reverse) Exon 4- | | (Reverse) Exon 3- | | (Reverse) Exon 2- | | (Reverse) Exon 1 - | | (Reverse) TATA Box - | |
| *Phyllostomus hastatus* | Start | End | Start | End | Start | End | Start | End | Start | End | Start | End |
|  | 5392096 | 5392091 | 5392319 | 5392153 | 5392987 | 5392916 | 5393236 | 5393129 | 5394249 | 5394057 | 5394295 | 5394289 |
|  | Length | 6 | Length | 84 | Length | 72 | Length | 108 | Length | 201 | Length | 7 |
| PVIA01018809.1 | (Forward) TATA Box | | (Forward) Exon 1 + | | (Forward) Exon 2 + | | (Forward) Exon 3 + | | (Forward) Exon 4 + | | (Forward) PolA + | |
| *Tonatia saurophila* | Start | End | Start | End | Start | End | Start | End | Start | End | Start | End |
|  | 3848 | 3854 | 3894 | 4094 | 4880 | 4987 | 5129 | 5200 | 5786 | 5906 | 5946 | 5951 |
|  | Length | 7 | Length | 201 | Length | 108 | Length | 72 | Length | 120 | Length | 6 |
| PVIA01020748.1 | (Reverse) PolA – | | Reverse) Exon 4- | | (Reverse) Exon 3- | | (Reverse) Exon 2- | | (Reverse) Exon 1 - | | (Reverse) TATA Box - | |
| *Tonatia saurophila* | Start | End | Start | End | Start | End | Start | End | Start | End | Start | End |
|  | 3709 | 3704 | 3874 | 3749 | 4543 | 4472 | 4792 | 4685 | 5796 | 5596 | 5842 | 5836 |
|  | Length | 7 | Length | 126 | Length | 72 | Length | 108 | Length | 201 | Length | 7 |
| PVIA01020748.1 | (Reverse) PolA – | | (Reverse) Exon 4- | | (Reverse) Exon 3- | | (Reverse) Exon 2- | | (Reverse) Exon 1 - | | (Reverse) TATA Box - | |
| *Phyllostomus hastatus* | Start | End | Start | End | Start | End | Start | End | Start | End | Start | End |
|  | 3709 | 3704 | 3874 | 3749 | 4543 | 4472 | 4792 | 4685 | 5796 | 5596 | 5842 | 5836 |
|  | Length | 6 | Length | 84 | Length | 72 | Length | 108 | Length | 201 | Length | 7 |
| NW_023512848.1 | (Reverse) PolA – | | (Reverse) Exon 4- | | (Reverse) Exon 3- | | (Reverse) Exon 2- | | (Reverse) Exon 1 - | | (Reverse) TATA Box - | |
| *Sturnira hondurensis* | Start | End | Start | End | Start | End | Start | End | Start | End | Start | End |
|  | 20683712 | 20683717 | 20683852 | 20683757 | 20684521 | 20684450 | 20684771 | 20684664 | 20685810 | 20685610 | 20685856 | 20685850 |
|  | Length | 6 | Length | 96 | Length | 72 | Length | 108 | Length | 201 | Length | 7 |
| VMDR010105903.1 | (Forward) TATA Box | | (Forward) Exon 1 + | | (Forward) Exon 2 + | | (Forward) Exon 3 + | | (Forward) Exon 4 + | | (Forward) PolA + | |
| VMDR010252616.1* | Start | End | Start | End | Start | End | Start | End | Start | End | Start | End |
| VMDR010000046.1** | 2474 | 2480 | 2520 | 2720 | 45 | 152 | 283* | 354* | 823** | 930** | 971** | 976** |
| *Macrotus californicus* | *Length* | *7* | *Length* | *201* | *Length* | *108* | *Length* | *72* | *Length* | *120* | *Length* | *6* |
| JAPYXV010000007.1 | (Reverse) PolA – | | (Reverse) Exon 4- | | (Reverse) Exon 3- | | (Reverse) Exon 2- | | (Reverse) Exon 1 - | | (Reverse) TATA Box - | |
| *Trachops cirrhosus* | Start | End | Start | End | Start | End | Start | End | Start | End | Start | End |
|  | 4710435 | 4710430 | 4710580 | 4710473 | 4711240 | 4711169 | 4711488 | 4711381 | 4712314 | 4712114 | 4712359 | 4712353 |
|  | Length | 7 | Length | 96 | Length | 72 | Length | 108 | Length | 201 | Length | 7 |
| JAPYXV010000007.1 | (Reverse) PolA – | | (Reverse) Exon 4- | | (Reverse) Exon 3- | | (Reverse) Exon 2- | | (Reverse) Exon 1 - | | (Reverse) TATA Box - | |
| *Trachops cirrhosus* | Start | End | Start | End | Start | End | Start | End | Start | End | Start | End |
|  | 473736 | 473731 | 473889 | 473776 | 474552 | 474481 | 474800 | 474693 |  |  |  |  |
|  | Length | 6 | Length | 111 | Length | 72 | Length | 108 | Length | ? | Length | ? |
| JAPYXV010000007.1 | (Reverse) PolA – | | (Reverse) Exon 4- | | (Reverse) Exon 3- | | (Reverse) Exon 2- | | (Reverse) Exon 1 - | | (Reverse) TATA Box - | |
| *Trachops cirrhosus* | Start | End | Start | End | Start | End | Start | End | Start | End | Start | End |
|  | 4225 | 4220 | 4377 | 4264 | 5040 | 4969 | 5288 | 5181 | 6111 | 5911 | 6156 | 6150 |
|  | Length | 6 | Length | 114 | Length | 72 | Length | 108 | Length | 201 | Length | 7 |

**Table S2.** Summary of the NCBI reference sequences of the genomic DNA (gDNA) utilized for the mining process. The precise positions of the cathelicidins' untranslated regions (UTRs) and exon DNA sequences, oriented in the forward or reverse direction, are delineated using distances measured in base pairs (bp) from the beginning of the chromosome, contig, or scaffold employed. Additionally, the length of each sequence is indicated in base pairs (bp). The symbol (?) signifies an incomplete gDNA sequence.

| **Reference Sequences** | | **Cathelicidins' genomic location (base pair length)** | | | | | | | | | | | |
| --- | --- | --- | --- | --- | --- | --- | --- | --- | --- | --- | --- | --- | --- |
| JAPYXV010022013.1 | | (Reverse) PolA – | | (Reverse) Exon 4- | | (Reverse) Exon 3- | | (Reverse) Exon 2- | | (Reverse) Exon 1 - | | (Reverse) TATA Box - | |
| *Trachops cirrhosus* | | Start | End | Start | End | Start | End | Start | End | Start | End | Start | End |
|  |  | 2268 | 2264 | 2417 | 2310 | 3037 | 3006 | 3283 | 2318 |  |  |  |  |
|  |  | Length | 6 | Length | 108 | Length | 72 | Length | Incomplete | Length | ? | Length | ? |
| JAPYXV010039841.1 | | (Reverse) PolA – | | (Reverse) Exon 4- | | (Reverse) Exon 3- | | (Reverse) Exon 2- | | (Reverse) Exon 1 - | | (Reverse) TATA Box - | |
| *Trachops cirrhosus* | | Start | End | Start | End | Start | End | Start | End | Start | End | Start | End |
|  |  | 700 | 696 | 848 | 741 | 1512 | 1441 |  |  |  |  |  |  |
|  |  | Length | 6 | Length | 108 | Length | 72 | Length | ? | Length | 201 | Length | ? |
| JAPYXV010039841.1 | | (Reverse) PolA – | | (Reverse) Exon 4- | | (Reverse) Exon 3- | | (Reverse) Exon 2- | | (Reverse) Exon 1 - | | (Reverse) TATA Box - | |
| *Trachops cirrhosus* | | Start | End | Start | End | Start | End | Start | End | Start | End | Start | End |
|  |  | 5646 | 5641 | 5793 | 5686 | 6461 | 6390 | 6700 | 6602 |  |  |  |  |
|  |  | Length | 6 | Length | 108 | Length | 72 | Length | Incomplete | Length | ? | Length | ? |
| NW_023416307.1 | | (Forward) TATA Box | | (Forward) Exon 1 + | | (Forward) Exon 2 + | | (Forward) Exon 3 + | | (Forward) Exon 4 + | | (Forward) PolA + | |
| *Rousettus aegyptiacus* | | Start | End | Start | End | Start | End | Start | End | Start | End | Start | End |
|  |  | 154681295 | 154681301 | 154681342 | 154681542 | 154682117 | 154682224 | 154682374 | 154682445 | 154683057 | 154683179 | 154683226 | 154683231 |
|  |  | Length | 7 | Length | 201 | Length | 108 | Length | 72 | Length | 123 | Length | 6 |
| NW_023416307.1 | | (Forward) TATA Box | | (Forward) Exon 1 + | | (Forward) Exon 2 + | | (Forward) Exon 3 + | | (Forward) Exon 4 + | | (Forward) PolA + | |
| *Cynopterus brachyotis* | | Start | End | Start | End | Start | End | Start | End | Start | End | Start | End |
|  |  | 68387 | 68393 | 68434 | 68634 | 69197 | 69304 | 69454 | 69525 | 70158 | 70292 | 70333 | 70338 |
|  |  | Length | 7 | Length | 201 | Length | 108 | Length | 72 | Length | 135 | Length | 6 |
| PVKZ01005166.1 | | (Forward) TATA Box | | (Forward) Exon 1 + | | (Forward) Exon 2 + | | (Forward) Exon 3 + | | (Forward) Exon 4 + | | (Forward) PolA + | |
| *Macroglossus sobrinus* | | Start | End | Start | End | Start | End | Start | End | Start | End | Start | End |
|  |  | 43675 | 43681 | 43722 | 43922 | 44512 | 44619 | 44765 | 44836 | 45457 | 45591 | 45637 | 45042 |
|  |  | Length | 7 | Length | 201 | Length | 108 | Length | 72 | Length | 135 | Length | 6 |
| CM053507.1 | | (Forward) TATA Box | | (Forward) Exon 1 + | | (Forward) Exon 2 + | | (Forward) Exon 3 + | | (Forward) Exon 4 + | | (Forward) PolA + | |
| **Chromosome 7** | | Start | End | Start | End | Start | End | Start | End | Start | End | Start | End |
| *Eidolon dupreanum* | | 148669045 | 148669052 | 148669092 | 148669292 | 148669828 | 148669935 | 148670078 | 148670149 | 148670747 | 148670881 | 148670925 | 148670930 |
|  |  | Length | 7 | Length | 201 | Length | 108 | Length | 72 | Length | 135 | Length | 6 |
| KE779879.1 | | (Forward) TATA Box | | (Forward) Exon 1 + | | (Forward) Exon 2 + | | (Forward) Exon 3 + | | (Forward) Exon 4 + | | (Forward) PolA + | |
| *Eidolon helvum* | | Start | End | Start | End | Start | End | Start | End | Start | End | Start | End |
|  |  | 16558 | 16565 | 16605 | 16805 | 17387 | 17494 | 17637 | 17708 | 18308 | 18442 | 18483 | 18488 |
|  |  | Length | 7 | Length | 201 | Length | 108 | Length | 72 | Length | 135 | Length | 6 |
| NW_024349803.1 | | (Forward) TATA Box | | (Forward) Exon 1 + | | (Forward) Exon 2 + | | (Forward) Exon 3 + | | (Forward) Exon 4 + | | (Forward) PolA + | |
| *Pteropus giganteus* | | Start | End | Start | End | Start | End | Start | End | Start | End | Start | End |
|  |  | 19806297 | 19806302 | 19806344 | 19806544 | 19807134 | 19807241 | 19807391 | 19807462 | 19808086 | 19808199 | 19808240 | 19808245 |
|  |  | Length | 7 | Length | 201 | Length | 108 | Length | 72 | Length | 114 | Length | 6 |
| NC_046300.1 | | (Reverse) PolA – | | (Reverse) Exon 4- | | (Reverse) Exon 3- | | (Reverse) Exon 2- | | (Reverse) Exon 1 - | | (Reverse) TATA Box - | |
| **Chromosome 17** | | Start | End | Start | End | Start | End | Start | End | Start | End | Start | End |
| *R. ferrumequinum* | | 7778944 | 7778939 | 7779122 | 7778985 | 7779780 | 7779709 | 7780028 | 7779921 | 7780857 | 7780660 | 7780904 | 7780898 |
|  |  | Length | 6 | Length | 141 | Length | 72 | Length | 108 | Length | 198 | Length | 7 |
| NW_017738954.1 | | (Reverse) PolA – | | (Reverse) Exon 4- | | (Reverse) Exon 3- | | (Reverse) Exon 2- | | (Reverse) Exon 1 - | | (Reverse) TATA Box - | |
| *Rhinolophus sinicus* | | Start | End | Start | End | Start | End | Start | End | Start | End | Start | End |
|  |  | 2535389 | 2535384 | 2535569 | 2535429 | 2536226 | 2536155 | 2536462 | 2536462 | 2537315 | 2537118 | 2537362 | 2537536 |
|  |  | Length | 6 | Length | 141 | Length | 72 | Length | 117 | Length | 198 | Length | 7 |
| NW_017738954.1 | | (Reverse) PolA – | | (Reverse) Exon 4- | | (Reverse) Exon 3- | | (Reverse) Exon 2- | | (Reverse) Exon 1 - | | (Reverse) TATA Box - | |
| *Hipposideros armiger* | | Start | End | Start | End | Start | End | Start | End | Start | End | Start | End |
|  |  | 2291019 | 2291014 | 2291194 | 2291060 | 2291844 | 2291773 | 2292104 | 2291979 | 2292948 | 2292751 | 2292995 | 2292989 |
|  |  | Length | 6 | Length | 135 | Length | 72 | Length | 126 | Length | 198 | Length | 7 |
| NW_017738954.1 | | (Reverse) PolA – | | (Reverse) Exon 4- | | (Reverse) Exon 3- | | (Reverse) Exon 2- | | (Reverse) Exon 1 - | | (Reverse) TATA Box - | |
| *Hipposideros armiger* | | Start | End | Start | End | Start | End | Start | End | Start | End | Start | End |
|  |  | 2291019 | 2291014 | 2291194 | 2291060 | 2291844 | 2291773 | 2292104 | 2291979 | 2292948 | 2292751 | 2292995 | 2292989 |
|  |  | Length | 6 | Length | 135 | Length | 72 | Length | 126 | Length | 198 | Length | 7 |
| JAHQIX010028625.1 | | (Reverse) PolA – | | (Reverse) Exon 4- | | (Reverse) Exon 3- | | (Reverse) Exon 2- | | (Reverse) Exon 1 - | | (Reverse) TATA Box - | |
| *Hipposideros armiger* | | Start | End | Start | End | Start | End | Start | End | Start | End | Start | End |
|  |  | 7662428 | 7662433 | 7662608 | 7662474 | 7663254 | 7663183 | 7663517 | 7663392 | 7664361 | 7664164 | 7664408 | 7664402 |
|  |  | Length | 6 | Length | 135 | Length | 72 | Length | 126 | Length | 198 | Length | 7 |
| NW_023416307.1 | | (Forward) TATA Box | | (Forward) Exon 1 + | | (Forward) Exon 2 + | | (Forward) Exon 3 + | | (Forward) Exon 4 + | | (Forward) PolA + | |
| *Hipposideros galeritus* | | Start | End | Start | End | Start | End | Start | End | Start | End | Start | End |
|  |  | 6569 | 6575 | 6616 | 6813 | 7463 | 7588 | 7723 | 7794 | 8379 | 8513 | 8554 | 8559 |
|  |  | Length | 7 | Length | 201 | Length | 108 | Length | 72 | Length | 135 | Length | 6 |
| PUFA01000145.1 | | (Forward) TATA Box | | (Forward) Exon 1 + | | (Forward) Exon 2 + | | (Forward) Exon 3 + | | (Forward) Exon 4 + | | (Forward) PolA + | |
| *Eonycteris spelaea* | *Non-functional* | Start | End | Start | End | Start | End | Start | End | Start | End | Start | End |
|  |  | 1822316 | 1822421 | 1823128 | 1822421 | 1823128 | 1823245 | 1823395 | 1823466 | 1824067 | 1824204 | 1824244 | 1824249 |
|  |  | Length | 5 | Length | 60 | Length | 108 | Length | 72 | Length | 138 | Length | 6 |

**Table S2.** Summary of the NCBI reference sequences of the genomic DNA (gDNA) utilized for the mining process. The precise positions of the cathelicidins' untranslated regions (UTRs) and exon DNA sequences, oriented in the forward or reverse direction, are delineated using distances measured in base pairs (bp) from the beginning of the chromosome, contig, or scaffold employed. Additionally, the length of each sequence is indicated in base pairs (bp). The symbol (?) signifies an incomplete gDNA sequence.
