# Supplementary material for: Genome Mining and Structural Study of Cathelicidins Across Chiroptera Species
Source: Biochem Res Int. 2025 Sep 23;2025:5461549. doi: 10.1155/bri/5461549 (PMC12483743; doi:10.1155/bri/5461549)
Supplement: Supporting Information 7 — Table S3: Incomplete cathelicidin sequences from three bat species displayed scaffolds that were either incomplete or comprised of ambiguous ‘N' bases. [file 5461549.f7.docx]

| NCBI Reference Sequence | Families | Species | **Sequences**  _______________________________1^st^ Exon________________________________________________________________2^nd^ Exon_____________________________3^rd^ Exon__________---------------------------------4^th^ Exon----------------------------------------------- |
| --- | --- | --- | --- |
| NW_023416317.1 | ***Vespertilionidae*** | ***Myotis myotis*** | **MATQRNSLCWGRWPLLLLLLGLAMPLPPAAARALSYQEAVRLAVQGFNQRSREASLYRLLQQDPQPQG GEPSGHPTRITSTVCSATSLPGRSGPGPDR** |
| ELK24989.1 |  | ***Myotis davidii*** | **LVKVCVGTVTLDQDNGSYDVVCEEIKDVILGAENLGERIKNAKKKVWEKIKSFGRRIKEFFRKPSPEVEP** |
| ELK24989.1 |  | ***Myotis davidii*** | **MEAQRNSLCGGRWPLLLLLLGLAMPWPPAAARALSYQEAVGLAVQGFNQRSREASLYRLLQQDPQPQGDLNPDTPKPVSFTLKETVCPRTTRQPPEQCDFKENGLVKACAGTVTLDQDTGSYDVVCEG** |
| JAPYXV010039841.1 | ***Phyllostomidae*** | ***Trachops cirrhosus*** | **LVKQCVGTVTLDQANSYFDINCAEIQDVRLGRRFQRFGRRFRRLLDRIRPRPPMVPGFA** |
| JAPYXV010011263.1 |  | ***Trachops cirrhosus*** | **PNTPKPVSFTLKETVCPRTTQLPPEQCEFKENGLVKQCVGTVTLGEAKGDLDIDCAEIQDVGLRRGLRKVGRAIGGLLRRTHIHVHVGFPFG** |
| JAPYXV010022013.1 |  | ***Trachops cirrhosus*** | **KETVCPRTTQLPPEQCEFKENGLVKQCVGTVTLDQANGYFDINCAEIQDVGLGSRFQRFGRRIRRLLERFRPRPPMVTGFA** |
| JAPYXV010000007.1 |  | ***Trachops cirrhosus*** | **DDNPNTPKPVSFTLKETVCPRTTQLPPEQCEFKENGLVKQCVGTVTLGEAKGYFDIDCAEIQDVVVAPLVRIGGRIVGKIAGEVIKRQYEKHRQNRG** |
| NCBI Reference Sequence | Families | Species | **Observations** |
| NW_023416317.1 | ***Vespertilionidae*** | ***Myotis myotis*** | The second and fourth exons are absent or incomplete. N-filled scaffold. |
| ELK24989.1 |  | ***Myotis davidii*** | The first and second exons are missing. N-filled scaffold. |
| ELK24989.1 |  | ***Myotis davidii*** | Scaffold full of N, cannot find the 4th exon |
| JAPYXV010039841.1 | ***Phyllostomidae*** | ***Trachops cirrhosus*** | Missing the first and second exons, the scaffold is incomplete. |
| JAPYXV010011263.1 |  | ***Trachops cirrhosus*** | Missing the first exon and a portion of the second, the scaffold is incomplete. |
| JAPYXV010022013.1 |  | ***Trachops cirrhosus*** | Missing the first exon and a portion of the second, the scaffold is incomplete. |
| JAPYXV010000007.1 |  | ***Trachops cirrhosus*** | Scaffold with N-filled regions where the first exon is expected to be located. |

**Table S3.** Incomplete cathelicidin sequences from three bat species displayed scaffolds that were either incomplete or comprised of ambiguous 'N' bases.
